# Impact of frailty, mild cognitive impairment and cognitive frailty on adverse health outcomes among community-dwelling older adults: A systematic review and meta-analysis

**DOI:** 10.3389/fmed.2022.1009794

**Published:** 2022-10-31

**Authors:** Baoyu Chen, Mingting Wang, Qin He, Yong Wang, Xiaoxing Lai, Hongguang Chen, Mengqian Li

**Affiliations:** ^1^Key Laboratory of Mental Health, Institute of Mental Health, Ministry of Health (Peking University), National Clinical Research Center for Mental Disorders, Peking University Sixth Hospital, Beijing, China; ^2^Department of Psychosomatic Medicine, The First Affiliated Hospital of Nanchang University, Nanchang, China; ^3^Peking Union Medical College Hospital, Beijing, China

**Keywords:** frailty, cognitive impairment, cognitive frailty, adverse outcomes, geriatric, meta-analysis

## Abstract

**Aims:**

This study analyzes the impact of frailty, mild cognitive impairment, and cognitive frailty on adverse outcomes in community-dwelling older adults.

**Methods:**

This systematic review and meta-analysis were conducted using the PRISMA guidelines and MOOSE statement. We developed a specific search strategy for each electronic database and searched PubMed, Web of Science, MEDLINE, and Embase from initial records to July 2021. The studies on adverse outcomes of frailty, pre-frailty, mild cognitive impairment, and mild cognitive impairment with pre-frailty and cognitive frailty were included. Two researchers independently extracted data based on a spreadsheet and assessed the risk of bias. The primary outcomes were mortality, dementia, disability, and hospitalization. The second outcome included quality of life and falls. All analysis was conducted by using Review Manager (RevMan) 5.3 software.

**Results:**

A total of 22 cohort studies (71,544 older adults with mean age ranging from 65.1 to 93.6 years) were included with a low risk of bias and high methodological quality with a NOS score ≥7. Compared to robust elders, individuals identified as frailty were associated with a higher risk of mortality (RR = 2.11, 95% CI: 1.57–2.83) and disability (RR = 5.91, 95% CI: 2.37–14.74). Mild cognitive impairment with pre-frailty was associated with mortality (RR = 1.74, 95% CI: 1.48–2.05) and dementia (RR = 4.15, 95% CI: 1.87–9.20). Pre-frailty was associated with a higher risk of mortality (RR = 1.29, 95% CI: 1.11–1.50). Cognitive frailty was associated with higher risk of incident mortality (RR = 2.41, 95% CI: 1.97–2.94), dementia (RR = 3.67, 95% CI: 2.81–4.78), disability (RR = 11.32, 95% CI: 4.14–30.97), and hospitalization (RR = 2.30, 95% CI: 1.60–3.32), as well as poor quality of life.

**Conclusion:**

Cognitive frailty could be a comprehensive psychosomatic predictor for adverse outcomes among older people. Interactions between frailty, mild cognitive impairment, and cognitive frailty on adverse outcomes must be further explored.

**Systematic review registration:**

[https://inplasy.com/inplasy-2022-5-0064/], identifier [INPLASY202250064].

## Introduction

Epidemiologic studies showed that the proportion of the population over 60 years is expected to double from 12 to 22% globally between 2015 and 2050 (1). Aging is a progressive and inevitable process of biology over time, manifested by degenerative changes in structure, a decline in function, and a weakening of adaptability and resistance (2, 3). As a result, the aging population increases rapidly, increasing the number of adults with frailty and mild cognitive impairment (4), putting tremendous pressure on the healthcare systems. Frailty and mild cognitive impairment are two critical indicators of the aging process (5).

Frailty has been described as a multidimensional clinical gerontological syndrome (6, 7). A cumulative decline of multiple physiological systems leads to reduced energy reserves, increased susceptibility to stressors, and dysregulated physiological system dynamic balance (8, 9). There are various screening methods for the assessment of frailty syndrome, and most studies have been based on the Fried phenotypic that was proposed by Fried: fatigue or self-reported exhaustion, involuntary weight loss, weakness (poor grip strength), slow walking speed, and lack of physical activity. Frailty is defined as the presence of at least three of these conditions, with one or two being defined as pre-frailty, and if none of these is non-frailty (10). Pre-frailty is an intermediate stage between non-frailty and frailty, and it may be a temporary state in which some older adults can improve muscle strength and regain energy following exercise. However, many older adults with pre-frailty experience continued physical decline, increasing the risk of mortality, and over time, pre-frailty may progress to frailty. A systematic review showed that frailty in community-dwelling was 10.7% (11).

Mild cognitive impairment is described as a decline of intellectual functions such as memory, thinking, and executive function, which is characterized by a moderate decline in one or more cognitive areas at previous levels, but not within the range of dementia (12). The American Academy of Neurology (AAN) guideline noted that the prevalence of mild cognitive impairment in older people over 60 ranged from 6.7 to 25.2% and increased with age (13). Data showed that mild cognitive impairment was significantly associated with frailty (14, 15). The International Academy on Nutrition and Aging (IANA) and the International Association of Gerontology and Geriatrics (IAGG) provided the first definition of a ‘Cognitive Frailty’ condition in older adults, definition as the coexistence of frailty and mild cognitive impairment (Clinical Dementia Rating [CDR] = 0.5), excluding concurrent Alzheimer’s disease or other types of dementia (16). Cognitive frailty includes potentially reversible cognitive frailty and reversible cognitive frailty. The former is indicated by mild cognitive impairment (MCI) (CDR = 0.5), and the latter by a pre-MCI subjective cognitive decline (SCD) (CDR = 0) and positive biomarkers of neurodegeneration (17). A meta-analysis showed that the estimated prevalence of cognitive frailty was 6% in older people community-dwelling (7). Recognition of associations between predictors as mild cognitive impairment and frailty alone and combined, and multiple adverse health outcomes, could not only inform treatment decisions and goals of care, but also provide predictors for early identification and intervention among the increasing old populations to reduce the occurrence of adverse health outcomes (18).

Previous systematic reviews and meta-analyses have shown that the coexistence of physical frailty and mild cognitive impairment can detect a cumulative negative effect, significantly increasing all-cause mortality or dementia (19–21). However, those have focused on the association of cognitive frailty with all-cause mortality and dementia, with relatively single adverse outcomes. Furthermore, findings remain controversial due to the differences in sample size, study design, measurement, the definition of frailty and adverse outcomes, and the population surveyed. Nevertheless, we hypothesized that frailty, mild cognitive impairment, and cognitive frailty would increase older people’s risk of adverse outcomes. Thus, this systematic review and meta-analysis were designed to explore the associations between individuals with frailty alone, mild cognitive impairment alone, cognitive frailty, and multiple adverse outcomes, offering evidence for further intervention.

## Methods

We reviewed studies assessing the effect of frailty, mild cognitive impairment, and cognitive frailty on adverse outcomes. The content of this systematic review followed the protocol of the Preferred Reporting Items for Systematic Review and Meta-analyses (PRISMA) guidelines and the Meta-Analysis of Observational Studies in Epidemiology (MOOSE) statement (22, 23) (see Supplementary Material 1). It was registered on the INPLASY website (INPLASY registration number: INPLASY202250064) (see Supplementary Material 2).

### Search strategy

We searched the relevant studies about the effect of frailty, mild cognitive impairment, and cognitive frailty on adverse outcomes (such as mortality, dementia, hospitalization, and disability) in older adults from PubMed, Web of Science, MEDLINE, Embase from initial records to July 2021. The search included selected keywords, medical subject headings, titles/abstracts, and free words, and these retrieval words were combined with Boolean operators (see Supplementary Material 3: S1). In parallel, the citation lists of relevant articles and reviews were screened to identify additional eligible articles which might have been missed by electronic search.

### Inclusion criteria and exclusion criteria

Inclusion criteria: (a) population: ≥60 years old in community-dwelling older adults; (b) intervention: participants with cognitive frailty, mild cognitive impairment with frailty, physical frailty (or pre-frailty), and mild cognitive impairment without dementia; (c) clinical diagnostic criteria: frailty, pre-frailty, mild cognitive impairment, cognitive frailty, and mild cognitive impairment with the pre-frailty need to be defined using internationally agreed-upon diagnostic criteria or need to describe specific diagnostic criteria; (d) comparison: robust older adults without mild cognitive impairment or physical frailty (or pre-frailty); (e) outcomes: reported the hazard ratio (HR), the odds ratio (OR), or the risk ratio (RR) with 95% confidence interval (CI) of the adverse outcomes, as well as outcomes or underlying data that contribute to the calculation of the above values; (f) study design: a prospective cohort study or population-based longitudinal studies. Exclusion criteria: (a) case reports, meeting reports, reviews or systematic reviews, meta-analysis; (b) no relevant outcomes data (HR, OR, and RR) available or insufficient statistics; (c) non-English literature.

### Study selection and data extraction

The two investigators completed the entire work independently, and a third investigator participated in discussions to make a final decision if there was disagreement. Researchers independently searched titles and abstracts of relevant articles. If the information met the selection criteria, the full text was analyzed. The process was carried out strictly following the Preferred Reporting Items for Systematic Reviews and Meta-Analyses (PRISMA) guidance and the Meta-Analysis of Observational Studies in Epidemiology (MOOSE) statement. We used a spreadsheet to record information from eligible articles about the information on the author, publication year, country, sample size, frailty categories, average age, the definition of frailty/mild cognitive impairment (assessment tools), prevalence of frailty/mild cognitive impairment/cognitive frailty, follow-up time, adverse outcomes and effect measure (OR, RR, and HR). We also extracted data on pre-frailty and mild cognitive impairment with pre-frailty. When measures of effect with varying degrees of adjustment were provided, the most adjusted estimate was used for the present study.

### Quality evaluation

Two researchers independently scored the quality of the studies included in meta-analyses following the Newcastle-Ottawa scale (NOS) used for cohort and case-control studies. Any disagreements were discussed until a consensus was reached. The NOS scale consisted of nine criteria, covering three elements selection, comparability, and outcome: (1) selection: representativeness of the exposed cohort, selection of the non-exposed cohort, ascertainment of exposure, demonstration that outcome of interest was not present at the start of the study; (2) comparability: according to the most critical or another essential factor to choose control; (3) outcomes: assessment of outcome, follow-up long enough for outcomes to occur, adequacy of follow up of cohorts. The evaluation used the semi-quantitative principle of the star system; the highest score was nine stars. Studies with a NOS score ≥6 are considered high quality (24).

### Statistical analysis

We adopted a random-effects model if the heterogeneity test significantly detected statistical difference (*I*^2^ > 50%) or used a fixed-effects model. When the heterogeneity was high, the source would be explored further. Sensitivity analyses were performed to examine whether eliminating any single study influenced the pooled effect. Subgroup analyses were conducted according to the different health states and divided into five groups: cognitive frailty, mild cognitive impairment with pre-frailty, frailty, pre-frailty, and mild cognitive impairment. Besides, the publication bias was analyzed using a visual inspection of the funnel plots (25). All analysis was conducted using Review Manager (RevMan) 5.3 software; a *p*-value < 0.05 was considered statistically significant.

### Ethical

This research did not involve human and animal experimentation.

## Results

### Study selection

**FIGURE 1:**
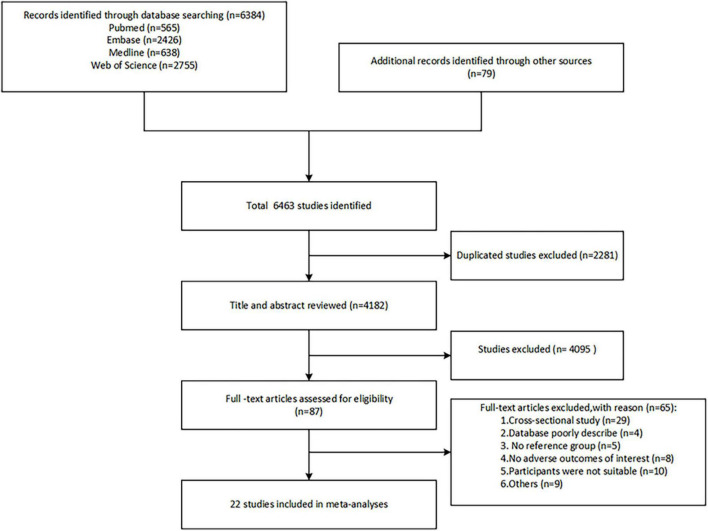
Flow diagram of the selection process of the studies.

A total of 6463 studies were identified by retrieving the four electronic databases and relevant meta-analyses of previous. Next, the 2281 duplicate studies were removed through automatic and manual checking, and after screening the titles and abstracts, 87 studies remained (see Figure 1). Finally, the full text was read according to the predefined inclusion and exclusion criteria, and 22 studies were finally included for meta-analysis.

### Study description

Twenty-two cohort studies involving 71,544 subjects were included in this study (see Supplementary Material 3: M1), of which 39,407 (55.1%) were female, and the mean age ranged from 65.1 to 93.6 years. The follow-up period was 2–14 years. All patients were recruited from communities such as the United States, France, Brazil, Mexico, Spain, Singapore, China, Korea, Canada, Japan, and Italy. The prevalence of frailty, mild cognitive impairment and cognitive frailty varied from 1.8–37.0, 2.5–55.6%, and 0.7–50.1%, respectively (Table 1).

**TABLE 1:** The characteristics of included studies.

Number	Author and Year	Country	Sample size/female (N)	Physical frailty categories (N)	Age (Mean ± SD)			
					Total	Robust	Pre-frailty	Frailty
1	(52)	United States	7338/4098	Robust: 6265 Frail: 1073	74.4 ± 7.0	73.2 ± 6.4	NR	Cognitively normal: 77.8 ± 7.6 Cognitively impaired: 79.9 ± 7.4
2	(27)	French	6030/3690	Robust: 2738 Pre-frail: 2871 Frail: 421	74.1 ± 5.2	73.5 ± 5.1	74.4 ± 5.2	76.6 ± 5.5
3	(33)	Brazil	405/231	Frail: 90	70.6 ± 7.1	NR	NR	NR
4	(26)	United States	639/378	Robust: 275 Pre-frail: 267	82.2 ± 3.7	81.8 ± 3.5	Cognitively intact: 82.3 ± 4.1 Cognitively impaired: 82.7 ± 4.3	NR
5	(26)	Mexico	1392/599	Robust: 574 Frail: 282	67.2 ± 6.1	66.6 ± 5.8	NR	68.2 ± 6.8
6	(53)	Spain	3677/2058	Robust: 1370 Pre-frail: 897	71.5 ± 7.8	68.8 ± 6.2	71.4 ± 7.7	NR
7	(31)	Singapore	2375/1515	Robust: 1552 Pre-frail: 792 Frail: 61	65.8 ± 7.5	64.2 ± 6.3	68.0 ± 8.3	75.3 ± 8.8
8	(30)	Singapore	1575/1021	Robust: 1044 Pre-frail: 502 Frail: 29	66.0 ± 7.6	64.6 ± 6.3	67.9 ± 8.2	75.9 ± 8.1
9	(11)	China	705/475	Robust: 86 Frail: 96	93.6 ± 3.3	92.5 ± 2.6	NR	93.1 ± 3.4
10	(51)	Taiwan, China	1103/510	Robust: 572 Dynapenia: 408	65.1 ± 9.5	61.1 ± 7.5	NR	68.5 ± 9.5
11	(51)	South Korea	11266/6726	Robust: 4654 Pre-frail: 5716 Frail: 896	72.9 ± 6.7	71.0 ± 5.4	73.9 ± 7.1	76.3 ± 7.3
12	(5)	Taiwan, China	678/314	Robust: 588	73.3 ± 5.3	73.0 ± 5.1	NR	NR
13	(5)	United States	690/451	Robust: 194	≥70	NR	NR	NR
14	(29)	Canada	252/158	Robust: 86 Pre-frail: 131 Frail: 35	76.7 ± 8.6	75.1 ± 7.0	76.7 ± 7.8	80.6 ± 13.2
15	(54)	Japan	5076/2963	Robust: 2374 Frail: 928	75.9 ± 6.9	NR	NR	NR
16	(55)	Japan	4570/2326	Robust: 2561 Frail: 752	71.9 ± 5.5	70.6 ± 4.5	NR	74.4 ± 6.3
17	(56)	Japan	4072/2087	Robust: 3601 Frail: 206	71.6 ± 5.2	71.1 ± 4.9	NR	76.9 ± 6.5
18	(57)	Italy	2373/1030	Robust: 2117 Frail: 172	76.7 ± 4.4	72.5 ± 5.3	NR	75.9 ± 4.9
19	(28)	Italy	2150/922	NR	73.2 ± 5.6	73.2 ± 5.6	NR	76.7 ± 4.6 (reversible cognitive frailty)
20	(45)	Canada	1751/1025	Robust: 1279 Frail: 472	77.5 ± 7.1	75.3 ± 6.2	NR	Cognitively intact: 79.0 ± 6.4 Cognitively impaired: 82.0 ± 7.5
21	(58)	Japan	9936/5139	Robust: 5274 Frail: 2250	73.5 ± 5.4	72.1 ± 4.7	NR	76.0 ± 5.7
22	(32)	China	3491/1691	Robust: 2008 Pre-frail: 1483	72.0 ± 4	NR	NR	NR

**Assessment method**		**Prevalence**						
**Frailty/ Per-frailty**	**Mild cognitive impairment**	**Frailty *N* (%)**	**Mild cognitive impairment *N* (%)**	**Cognitive frailty *N* (%)**	**Mild cognitive impairment with pre-frailty *N* (%)**	**Follow-up**	**Adverse outcome**	**Effect measure**

FP	HRS	1073 (14.6%)	1470 (20.0%)	397 (5.4%)	NR	8 years	Disability (ADL) Mortality	HR
FP	MMSE and IST	421 (7.0%)	711 (11.8%)	92 (1.5%)	345 (5.7%)	4 years	Disability (ADL, IADL, and Mobility) Hospitalization Mortality Dementia	OR/HR
FP	MMSE	90 (22.2%)	133 (32.8%)	44 (10.9%)	NR	4 years	Disability (IADL) Hospitalization Falls	OR
FP	MMSE	66 (16.9%)	178 (27.9%)	NR	81 (12.7%)	Frailty: 4 years Mortality: 10 years	Mortality Frailty	HR/OR
Self-reported responses based on six questions	CCCE	282 (22.0%)	246 (19.2%)	181 (13.0%)	NR	12 years	Mortality	HR
FS	MMSE	NR	1409 (38.3%)	832 (22.6%)	NR	14 years	Mortality Physical activity level	HR
FP	CMMSE	61 (2.6%)	476 (20.0%)	43 (1.8%)	212 (8.9%)	3 years	Disability (ADL, IADL) QOL (SF-12) Hospitalization Mortality	OR/HR
FP	CMMSE	29 (1.8%)	141 (9.0%)	15 (1.0%)	66 (4.2%)	3 years	Dementia	OR
FI	MMSE	96 (13.6%)	170 (24.1%)	353 (50.1%)	NR	4 years	Mortality	HR
Dynapenia	SPMSQ	408 (37.0%)	28 (2.5%)	95 (8.6%)	NR	4 years	Mortality	HR
Modified FP	MMSE-KC	896 (8.0%)	2855 (25.3%)	392 (3.3%)	1609 (13.8%)	3 years	Mortality	HR
Dynapenia (slowness and/ or weakness)	MMSE, CVVLT, BNT, VFT, CFT, DB, and CDT	NR	NR	90 (13.3%)	NR	2.5 years	Mortality	HR
FP	MMSE	NR	NR	45 (6.5%)	NR	11 years	Hospitalization Nursing Home Admission Disability (ADL, IADL, and mobility)	RR
FP	MoCA and CDR	35 (13.9%)	140 (55.6%)	27 (10.7%)	67 (26.6%)	5 years	Dementia Cognitive decline	HR
KCL (SR-MD, 5 items)	KCL (SR-CD, 3 items)	1686 (33.2%)	1774 (34.9%)	756 (14.9%)	NR	3 years	Mortality	HR
Slow walking speed or muscle weakness	NCGG-FAT	752 (16.5%)	676 (14.8%)	441 (9.6%)	NR	3 years	Dementia	HR
FP	NCGG-FAT	206 (5.1%)	222 (5.5%)	43 (1.1%)	NR	2 years	Dementia	HR
FP	MMSE	172 (7.2%)	67 (2.8%)	17 (0.7%)	NR	3.5 years	Disability (ADL) Mortality Dementia	RR
FP	MMSE and GDS-30	NR	NR	54 (2.5%)	NR	3.5 and 7 years	Dementia Mortality	HR
FI	MMSE	472 (27.0%)	537 (30.7%)	211 (12.1%)	NR	5 years	Mortality	HR
Walking-speed and grip-strength measurements	NCGG-FA	2250 (22.6%)	1303 (13.1%)	1109 (11.2%)	NR	2 years	Disability	HR
FP	CMMSE	NR	607 (17.4%)	NR	302 (8.7%)	Poor quality of life: 4 years Physical limitation: 4 years Hospitalization: 7 years Mortality: 12 years	Poor quality of life (SF-12) Physical limitation Hospitalization Mortality	OR

SD, standard deviations; FP, fried phenotype; FS, FRAIL Scale; FI, frailty index; MMSE, the Minimum Mental State Examination; CMMSE, the Cantonese version of Mini-Mental Status Examination/the Chinese version of the Mini-Mental State Examination; MMSE-KC, the Korean version of the Mini-Mental State Examination; MoCA, the Montreal Cognitive Assessment; HRS, the Health and Retirement Study; CDR, the Clinical Dementia Rating; IST, the Isaacs Set Test; NCGG-FAT, the National Center for Geriatrics and Gerontology-Functional Assessment Tool; KCI, the Kihon Checklist; SR-MD, self-reported mobility decline; SR-CD, self-reported cognitive decline; CCCE, the Cross-Cultural Cognitive Examination; CVVLT, the Chinese Version Verbal Learning Test; BNT, the Boston Naming Test; DB, the digital backward; VFT, the Verbal Fluency Test; CFT, the Taylor Complex Figure Test; CDT, the Clock Drawing Test; SPMSQ, the Short Portable Mental Status Questionnaire; GDS-30, the Geriatric Depression Scale-30 items; QOL, poor quality of life; SF-12, Short Form-12; ADL, activities of daily living; IADL, instrumental activities of daily living; HR, hazard ratio; OR, odds ratio; RR, rate ratio; NR, not reported.

#### Frailty, mild cognitive impairment, and cognitive frailty assessment

We found that frailty was most commonly defined by the Fried phenotype (FP) [the Cardiovascular Health Study criteria (CHS)], with 13 studies in which frailty met at least 3 of the five criteria: weight loss, exhaustion, low physical activity, slowness, and weakness. Of these, Downer et al. (26) used only four criteria of FP, including weight loss of more than 10 pounds, self-reported exhaustion, slow walking speed, and poor grip strength (pre-frail = 1 criterion, and frail = 2 + criteria) (26). In addition, one study used the FRAIL Scale (FS) assessment, and two used the frailty index (FI) to determine. Other assessment tools are not commonly used, such as walking speed and grip-strength measurements, dynapenia, the Kihon Checklist (KCL), and self-reported responses based on six questions.

To identify mild cognitive impairment, the most used was the Mini-Mental State Examination (MMSE), with 14 studies, but for cut-off varies slightly, from 18 to 27, or according to the level of education. Of these, Avila-Funes et al. (27) combined the Isaacs Set Test (IST) (27), and Solfrizzi et al. (28) combined the Geriatric Depression Scale-30 items (GDS-30) to determine mild cognitive impairment (28). In addition, three studies used the National Center for Geriatrics and Gerontology-Functional Assessment Tool (NCGG-FAT), and another one combined the Montreal Cognitive Assessment (MoCA) score below 26 and the Clinical Dementia Rating (CDR) of 0.5 to define mild cognitive impairment. Finally, the seldomly used ones include the Health and Retirement Study (HRS), the Cross-Cultural Cognitive Examination (CCCE), the Short Portable Mental Status Questionnaire (SPMQ), and KCL.

A definition of cognitive frailty was based on the coexistence of frailty and mild cognitive impairment in the original studies.

### Methodological quality

The methodological quality of the included 22 cohort studies was assessed using the NOS (see Supplementary Material 3: Table 1). The bias risk scores of all the reports ranged between 7 and 9 (a total score of 9), with six studies scoring full marks, ten studies scoring seven, and six studies scoring eight. The methodological quality of the included studies was high, with a low risk of bias. The primary bias for the included studies was that the follow-up was not long enough and the incomplete adjustment of important confounders in some articles. Hao et al. (11) was a community study on a 90 + year cohort in Sichuan Province in China (11). Montero-Odasso et al. (29) were done from geriatric clinics and a retirement community in London, Ontario (29). No stars were given for the representativeness of the exposed cohort for these two studies. Feng et al. (30) was an inadequacy of follow-up of cohorts, and therefore no star was given for this item (30).

### Primary outcomes

The results showed that individuals with cognitive frailty had a relatively high mortality risk, dementia, disability, and hospitalization (see Supplementary Material 3: Figure 1). The results displayed that cognitive frailty was the most effective predictor for mortality (RR = 2.41, 95% CI: 1.97–2.94), disability (RR = 11.32, 95% CI: 4.14–30.97), and hospitalization (RR = 2.30, 95% CI: 1.60–3.32) while mild cognitive impairment with pre-frailty was the strongest predictor for dementia (RR = 4.15, 95% CI: 1.87–9.20).

#### Mortality

**FIGURE 2:**
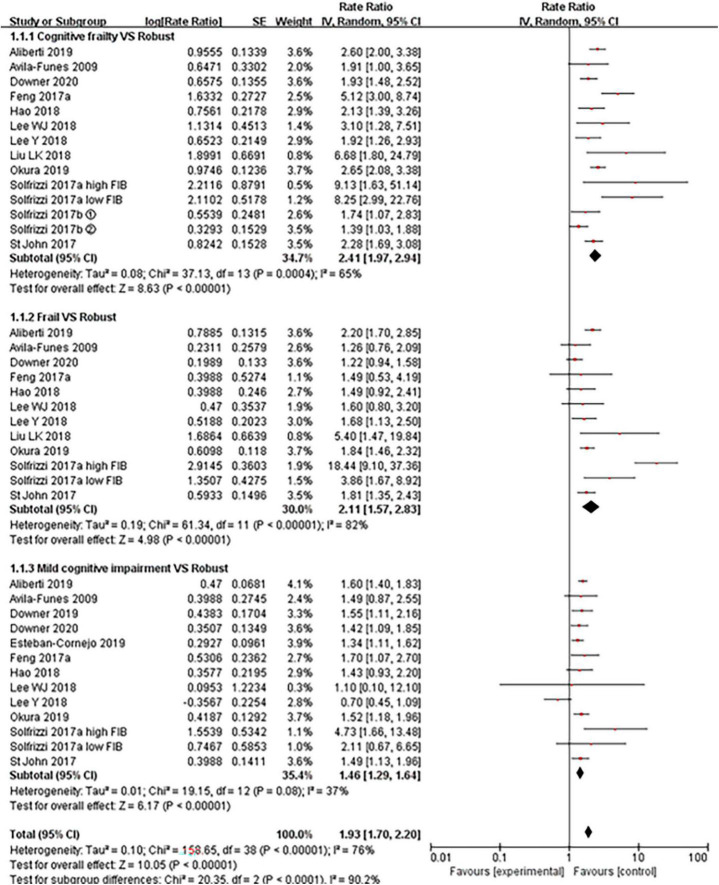
Forest plot of the association between cognitive frailty, frailty, and mild cognitive impairment and mortality in older adults (reference group: participants were free of frailty and mild cognitive impairment), using random-effects meta-analysis. ➀: follow-up 3.5 years; ➁: follow-up 7 years; 95% CI, 95% confidence interval; high FIB, high fibrinogen (inflammatory); low FIB, low fibrinogen (inflammatory).

Figure 2 depicted the relationships between cognitive frailty, frailty, mild cognitive impairment, and mortality, respectively. Compared to robust older adults, those with cognitive frailty had the highest risk of mortality (RR = 2.41, 95% CI: 1.97–2.94, *I*^2^ = 65%, *Z* = 8.63, *p* < 0.001), followed by the frailty group (RR = 2.11, 95% CI: 1.57–2.83, *I^2^* = 82%, *Z* = 4.98, *p* < 0.001), the mild cognitive impairment group (RR = 1.46, 95% CI: 1.29–1.64, *I*^2^ = 37%, *Z* = 6.17, *p* < 0.001). Mortality was significantly increased in all three subgroups. There was a stepwise association between the frailty category and mortality (see Supplementary Material 3: Figure 2). The RR for mortality was 1.74 (95% CI: 1.48–2.05, *I*^2^ = 0%, *Z* = 6.68, *p* < 0.001) for mild cognitive impairment with pre-frailty and 1.29 (95% CI: 1.11–1.50, *I^2^* = 0%, *Z* = 3.32, *p* < 0.001) for pre-frailty.

#### Dementia

**FIGURE 3:**
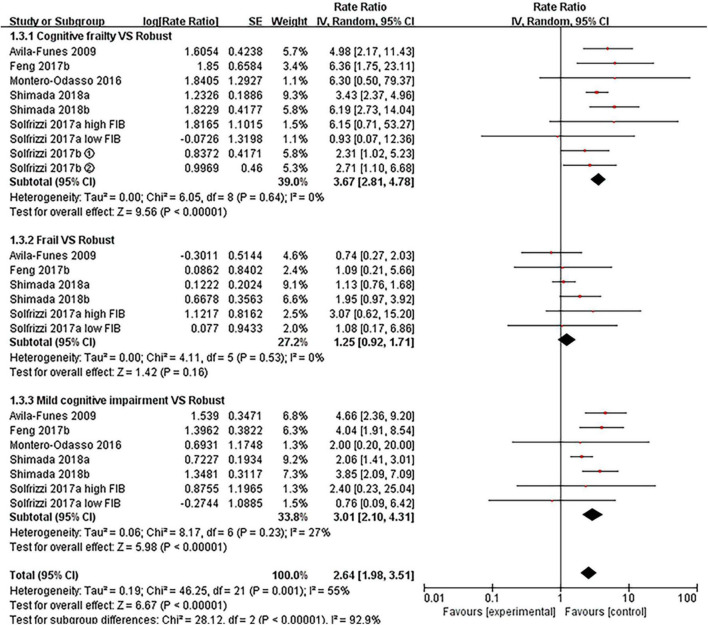
Forest plot of the association between cognitive frailty, frailty, and mild cognitive impairment and incident dementia in older adults (reference group: participants were free of frailty and mild cognitive impairment), using random-effects meta-analysis. ➀: follow-up 3.5 years; ➁: follow-up 7 years; 95% CI, 95% confidence interval; high FIB, high fibrinogen (inflammatory); low FIB, low fibrinogen (inflammatory).

Figure 3 described the relationships between cognitive frailty, frailty, mild cognitive impairment, mild cognitive impairment with pre-frailty, and pre-frailty (see Supplementary Material 3: Figure 3) with an incidence of dementia, respectively. In comparison to robust older people, the RR for dementia was 3.67 (95% CI: 2.81–4.78, *I*^2^ = 0%, *Z* = 9.56, *p* < 0.001) for cognitive frailty, 1.25 (95% CI: 0.92–1.71, *I*^2^ = 0%, *Z* = 1.42, *p* = 0.16) for frailty, 3.01 (95% CI: 2.10–4.31, *I*^2^ = 27%, *Z* = 5.98, *p* < 0.001) for mild cognitive impairment, 4.15 (95% CI: 1.87–9.20, *I*^2^ = 55%, *Z* = 3.51, *p* < 0.001) for mild cognitive impairment with pre-frailty, 1.00 (95% CI: 0.69–1.45, *I*^2^ = 5%, *Z* = 0.01, *p* = 0.99) for pre-frailty.

#### Disability

**FIGURE 4:**
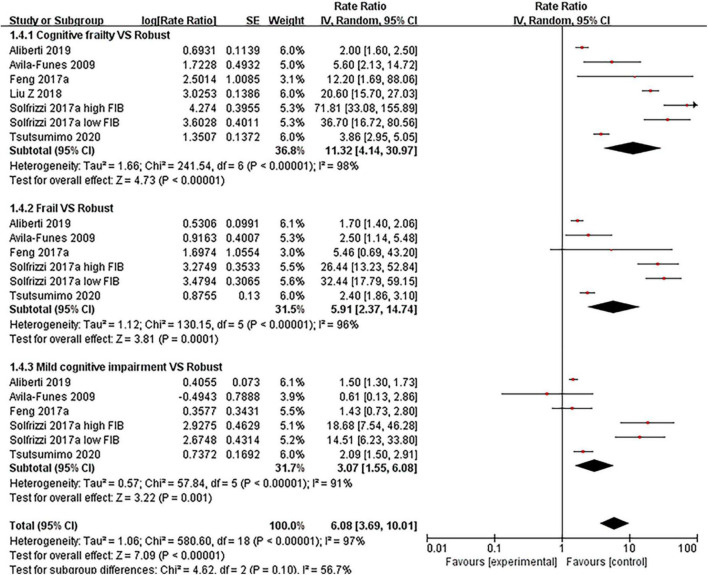
Forest plot of the association between cognitive frailty, frailty, and mild cognitive impairment and disability in older adults (reference group: participants were free of frailty and mild cognitive impairment), using random-effects meta-analysis. 95% CI, 95% confidence interval; high FIB, high fibrinogen (inflammatory); low FIB, low fibrinogen (inflammatory).

We examined the effect of cognitive frailty, frailty, mild cognitive impairment, and mild cognitive impairment with pre-frailty and pre-frailty on disability, respectively. The results showed that cognitive frailty was highly associated with disability compared with robust older people (RR = 11.32, 95% CI: 4.14–30.97, *I*^2^ = 98%, *Z* = 4.73, *p* < 0.001, see Figure 4), which was high than frailty (RR = 5.91, 95% CI: 2.37–14.74, *I*^2^ = 96%, Z = 3.81, *p* < 0.001) and mild cognitive impairment (RR = 3.07, 95% CI: 1.55–6.08, *I*^2^ = 91%, *Z* = 3.22, *p* < 0.001). Both included articles (27, 31) showed no statistically significant effects on disability in older people for both mild cognitive impairment with pre-frailty and pre-frailty.

#### Hospitalization

**FIGURE 5:**
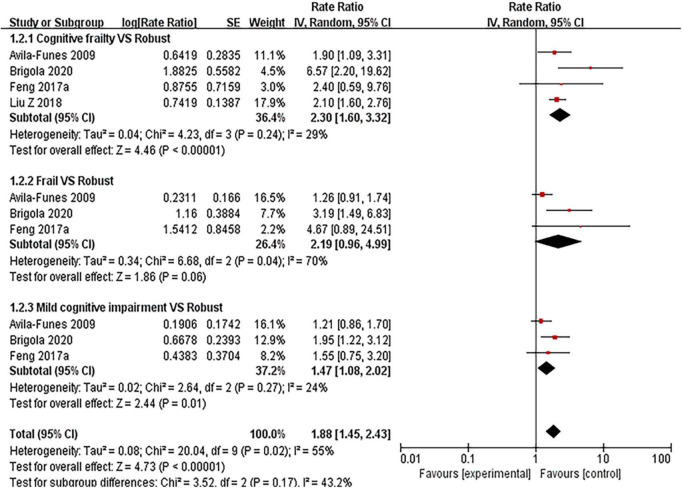
Forest plot of the association between cognitive frailty, frailty, and mild cognitive impairment and hospitalization in older adults (reference group: participants were free of frailty and mild cognitive impairment), using random-effects meta-analysis. 95% CI, 95% confidence interval.

Five articles (5, 27, 31–33) (see Figure 5 and Supplementary Material 3: Figure 4) involved hospitalization. Results showed that the risk for hospitalization with cognitive frailty had a significant increase (RR = 2.30, 95% CI: 1.60–3.32, *I*^2^ = 29%, *Z* = 4.46, *p* < 0.001) compared with those without. The RRs were 2.19 (95% CI: 0.96–4.99, *I*^2^ = 70%, *Z* = 1.86, *p* = 0.06) for frailty group and 1.47 (95% CI: 1.08–2.02, *I*^2^ = 24%, *Z* = 2.44, *p* = 0.01) for mild cognitive impairment group. The RRs were 1.20 (95% CI: 0.95–1.50, *I*^2^ = 42%, *Z* = 1.55, *p* = 0.12) in mild cognitive impairment with pre-frailty group. The two included articles (27, 31) showed an association between pre-frailty and risk of hospitalization in older people, with statistically significant results.

### Other adverse outcomes

#### Quality of life

Two studies (31, 32) used the Short Form-12 (SF-12) to evaluate the quality of life of the subjects. Feng et al. (31) showed that in comparison to robust older people, the OR for poor quality of life at baseline was 5.34 (95% CI: 2.42–11.77, *p* < 0.001) for cognitive frailty, 2.96 (95% CI: 1.05–8.31, *p* = 0.04), for frailty and 0.74 (95% CI: 0.50–1.10, *p* = 0.14) for mild cognitive impairment (31). After 3-year follow-up, cognitive frailty (OR = 26.9, 95% CI: 3.05–238.4, *p* = 0.03) appeared highly associated with poor quality of life compared to frailty (OR = 1.72, 95% CI: 0.29–10.03, *p* = 0.55) and mild cognitive impairment (OR = 0.82, 95% CI: 0.47–1.42, *p* = 0.47). Yu et al. (32) found that the older people who were mild cognitive impairment with pre-frailty were also associated with poor quality of life over the 4-year follow-up period (OR = 1.53, 95% CI: 1.06–2.22) (32).

#### Falls

Brigola et al. (33) evaluated the cumulative effect of mild cognitive impairment and frailty on falls in older adults over 4 years. Compared to robust older people, the OR was 1.11 (95% CI: 0.72–1.90) for mild cognitive impairment, 1.83 (95% CI: 0.83–3.95) for frailty, and 1.44 (95% CI: 0.51–4.05) for cognitive frailty (33). No cumulative effect regarding the occurrence of falls was found for any of the three clinically heterogeneous syndromes.

### Publication bias and sensitivity analysis

Given the rigorous design of the included studies, most funnel plots showed a basic symmetrical shape (see Supplementary Material 3: Figures 5–7). However, for a subgroup of disability (see Supplementary Material 3: Figure 8), its funnel plot showed asymmetrical, suggesting possible publication bias. In the sensitivity analysis of the primary adverse outcomes, we found a minor difference between the comprehensive effect value and the total comprehensive effect value after excluding each study, indicating that the results of this study were highly stable.

## Discussion

This systematic review and meta-analysis provided a pooled analysis of frailty, pre-frailty, mild cognitive impairment and cognitive frailty, and mild cognitive impairment with pre-frailty on adverse outcomes of older adults, with different effect values.

The results showed a higher risk of mortality and disability for individuals with frailty compared to robust older adults. The high correlation between frailty, mortality, and disability might relate to the fact that older adults often suffer from multiple chronic diseases and low levels of immune function. For example, cardiovascular disease, kidney disease, and diabetes, which overlap with frailty to create a vicious cycle, have been reported to be associated with mortality and contribute to a decline in their functioning, contributing to a detrimental effect on ADLs/IADLs (34–36). Meanwhile, frailty is often present in older people who are not yet disabled but might make them vulnerable to developing a disability in the presence of stressors (37). However, pre-frailty did not reach a statistically significant association with disability. A plausible explanation for this might be that pre-frailty is defined as the manifestation of only one or two frailty-related components with its severity not enough to meet the definition of ADLs/IADLs (38).

Furthermore, no association was observed between frailty and dementia or hospitalization. Although the current definition of frailty mainly focuses on physical aspects, whereas dementia is more of a neurocognitive disorder (39), frailty might not be a direct or sensitive predictor of dementia. Recently, a study indicated that individuals with a low degree of Alzheimer’s disease pathology might also be at risk for dementia if they have severe frailty, suggesting that frailty might mediate the association between brain neuropathology and impending dementia (40). Similarly, frailty was not found to be associated with hospitalization. According to the definition, frailty focuses on a slowly degraded and chronic state of physical function (10), easy to neglect by the patient and doctors, so it appears to be not associated with hospitalization. However, it also suggests the importance of including frailty in routine clinical assessments for early intervention, as it increases the risk of disability and mortality.

Mild cognitive impairment is a common clinical symptom seen in older people and is one of the independent risk factors of dementia. Mild cognitive impairment is not only a symptom but also a state of disease, which is accessible comorbid with many chronic physical and psychological diseases (41). This study exhibited that mild cognitive impairment was associated with mortality, dementia, disability, and hospitalization. Mild cognitive impairment was thought to be a possible age-related reduction in cognitive reserve, a physiological precursor to degenerative neurological disease (42). Older adults with mild cognitive impairment had a higher risk of dementia and disability than mortality and hospitalization. Mild cognitive impairment is an intermediate state of dementia, and older adults with mild cognitive impairment seem more likely to transition to clinical dementia (43). In contrast to frailty, which was only associated with mortality and disability—relatively more severe adverse outcomes, mild cognitive impairment was associated with all four adverse outcomes in older people, serving as a significant indicator for early identification of adverse outcomes.

Although frailty and mild cognitive impairment are usually regarded as two separate concepts, however, they tend to coexist in later life, interacting with each other and having cumulatively negative effects on health with significant adverse outcomes (21, 35, 44, 45). With mild cognitive impairment, older people tend to suffer from slow gait, fatigue, and reduced physical activity, increasing the risk of becoming frailty (14, 40). Bunce et al. (14) evaluated the association between specific cognitive domains and frailty and found that individuals classified as frailty at baseline showed deficits on tests that assessed information processing speed and verbal fluency (14). Results from prospective cohort studies also showed that people with mild cognitive impairment at baseline were more likely to develop frailty, influencing the trajectory of frailty and vice versa (15, 46). Nevertheless, it is also reported that there was a reverse association between cognitive frailty and adverse outcomes and that prodromal symptom of adverse outcomes might co-occur with or lead to cognitive frailty (30). In summary, cognitive frailty, a combination of frailty and mild cognitive impairment, was a more comprehensive assessment indicator for early identification and intervention for adverse outcomes in older people.

Our results suggested that frailty, mild cognitive impairment, cognitive frailty, pre-frailty, and mild cognitive impairment with pre-frailty, were strongly associated with adverse outcomes in older adults. While pre-frailty is defined as an intermediate stage between non-frailty and frailty, we believe intervention strategies could provide opportunities for reversal, which might be the best time to intervene. Meanwhile, mild cognitive impairment could improve the predictive ability of frailty and pre-frailty for mortality, dementia, disability, and hospitalization. Therefore, it was suggested that adding mild cognitive impairment to the simultaneous assessment of frailty or pre-frailty might help to better predict adverse outcomes in older adults.

Epidemiological and clinical studies have now explored many mechanisms to explain the link between frailty and mild cognitive impairment in chronic inflammation, including oxidative stress, hormonal dysregulation, cardiovascular risk, epigenetic changes, hypothalamic-pituitary-adrenal (HPA) axis dysfunction, and mental health (47, 48). In addition, understanding the temporal relationship between mild cognitive impairment and frailty is vital to predicting the occurrence of another impairment. However, the specific mechanisms of this relationship have not been elucidated, calling for further research to understand the underlying interrelated pathophysiological mechanisms and the sequence between them. Since frailty is a dynamic process and cognitive decline is modifiable, cognitive frailty may also be a reversible clinical syndrome but with the preventable decline of accompanying functions (49–51). Furthermore, cognitive frailty is strongly associated with poor quality of life in older people. The frailty components, including fatigue, slow walking speed, and lack of physical activity, partially overlap with the definition of poor quality of life, severely affecting the physical functioning, energy, social functioning, and mental health of older people, and was even worse when combined with mild cognitive impairment. So, it is necessary to have effective strategies that target the prevention and management of frailty and mild cognitive impairment in the ageing population throughout the progress.

### Strengths and limitations

The strengths of this review lie in the comprehensive search of multiple electronic databases and hand-searching to perform a more comprehensive analysis of the relationship between frailty, mild cognitive impairment, and cognitive frailty and increased risk of adverse outcomes in older adults. Moreover, all studies were sifted, and data were extracted by two trained reviewers independently, providing a reliable overview of the evidence in this field. The overall quality of included studies in this review was high. Moreover, the results of the included studies were moderately adjusted for confounding factors, making the overall heterogeneity of this study not high.

However, some limitations did exist in this study. (a) The measurements for frailty and mild cognitive impairment varied in the included literature, which might increase the heterogeneity of the results. However, there is also a strong correlation between different measurements. (b) Although most studies have adjusted confounding factors, the numbers and types differ, affecting the results. (c) Subgroup analysis by reversible and potentially reversible cognitive frailty was not performed since only two related pieces of literature were included. (d) Unpublished gray literature was not included, and only English articles were retrieved.

### Future directions

The evaluation instrument for frailty and cognitive frailty varies, with no clear consensus on the best instrument used for clinical practice—even some studies have taken a self-report approach to identify frailty. However, there was evidence showing the association between frailty, mild cognitive impairment, and specific biomarkers; it is necessary to develop well-established instruments and derive precise biomarkers for frailty and mild cognitive impairment, to carry on special screening programs and therapeutic strategies. Moreover, the focus will be on the complex biological processes of underlying frailty. Finally, a large sample size, multi-center, and follow-up studies are needed to dynamically explore the long-term interaction effect of frailty, mild cognitive impairment, and cognitive frailty on adverse outcomes among older people.

## Conclusion

This systematic review and meta-analysis provided an evidence-based practice for the associations between adverse health outcomes and frailty, mild cognitive impairment, and cognitive frailty among older people. It is suggested that cognitive frailty tends to be a comprehensive critical predictor of adverse outcomes for older people. Therefore, multidimensional measures should be adopted to reduce the occurrence of adverse outcomes through early identification and intervention among the steadily increasing old populations.

## Data availability statement

The original contributions presented in the study are included in the article/Supplementary material, further inquiries can be directed to the corresponding author/s.

## Author contributions

BC and HC had the initial research idea, formulated the research questions, designed the study, contributed to designing the searches and the statistical analysis plan, writing the manuscript, and interpreting the findings. BC searched for published work, selected articles, and extracted and analyzed the data. MW and QH drafted the protocol and manuscript and performed the statistical analysis. YW and XL helped with searching for and data selection and extraction. All authors have agreed on the final manuscript and the decision to submit it for publication.

## References

[1] World Health Organization. *Ageing and Health.* (2018) Available Online at: https://www.who.int/news-room/fact-sheets/detail/ageing-and-health. (accessed April 30, 2022).

[2] da Costa JP Vitorino R Silva GM Vogel C Duarte AC Rocha-Santos T. A synopsis on aging-theories, mechanisms and future prospects. *Ageing Res Rev.* (2016) 29:90–112. 10.1016/j.arr.2016.06.005 27353257 PMC5991498

[3] Kocsisova Z Kornfeld K Schedl T. Rapid population-wide declines in stem cell number and activity during reproductive aging in C. Elegans. *Development.* (2019) 146:dev173195. 10.1242/dev.173195 30936182 PMC6503983

[4] Ambagtsheer RC Beilby JJ Visvanathan R Dent E Yu S Braunack-Mayer AJ. Should we screen for frailty in primary care settings? A fresh perspective on the frailty evidence base: a narrative review. *Prev Med.* (2019) 119:63–9. 10.1016/j.ypmed.2018.12.020 30594533

[5] Liu LK Chen CH Lee WJ Wu YH Hwang AC Lin MH Cognitive frailty and its association with all-cause mortality among community-dwelling older adults in Taiwan: results from I-Lan longitudinal aging study. *Rejuvenation Res.* (2018) 21:510–7. 10.1089/rej.2017.2038 29644921

[6] Li Y Liu M Miyawaki CE Sun X Hou T Tang S Bidirectional relationship between subjective age and frailty: a prospective cohort study. *BMC Geriatr.* (2021) 21:395. 10.1186/s12877-021-02344-1 34187378 PMC8244193

[7] Zhang T Ren Y Shen P Jiang S Yang Y Wang Y Prevalence and associated risk factors of cognitive frailty: A systematic review and meta-analysis. *Front Aging Neurosci.* (2021) 13:755926. 10.3389/fnagi.2021.755926 35153714 PMC8832102

[8] Sugimoto T Sakurai T Ono R Kimura A Saji N Niida S Epidemiological and clinical significance of cognitive frailty: a mini review. *Ageing Res. Rev.* (2018) 44:1–7. 10.1016/j.arr.2018.03.002 29544875

[9] Mehdizadeh D Hale M Todd O Zaman H Marques I Petty D Associations between anticholinergic medication exposure and adverse health outcomes in older people with frailty: a systematic review and meta-analysis. *Drugs Real World Outcomes.* (2021) 8:431–58. 10.1007/s40801-021-00256-5 34164795 PMC8605959

[10] Fried LP Tangen CM Walston J Newman AB Hirsch C Gottdiener J Frailty in older adults: evidence for a phenotype. *J Gerontol A Biol Sci Med Sci.* (2001) 56:M146–56. 10.1093/gerona/56.3.m146 11253156

[11] Hao Q Dong B Yang M Dong B Wei Y. Frailty and cognitive impairment in predicting mortality among oldest-old people. *Front Aging Neurosci.* (2018) 10:295. 10.3389/fnagi.2018.00295 30405390 PMC6201058

[12] Sachdev PS Lipnicki DM Crawford J Reppermund S Kochan NA Trollor JN Factors predicting reversion from mild cognitive impairment to normal cognitive functioning: a population-based study. *PLoS One.* (2013) 8:e59649. 10.1371/journal.pone.0059649 23544083 PMC3609866

[13] Petersen RC Lopez O Armstrong MJ Getchius TSD Ganguli M Gloss D Practice guideline update summary: mild cognitive impairment: report of the guideline development, dissemination, and implementation subcommittee of the American academy of neurology. *Neurology.* (2018) 90:126–35. 10.1212/WNL.0000000000004826 29282327 PMC5772157

[14] Bunce D Batterham PJ Mackinnon AJ. Long-term associations between physical frailty and performance in specific cognitive domains. *J Gerontol B Psychol Sci Soc Sci.* (2019) 74:919–26. 10.1093/geronb/gbx177 29401240

[15] Nari F Jang BN Youn HM Jeong W Jang SI Park EC. Frailty transitions and cognitive function among South Korean older adults. *Sci Rep.* (2021) 11:10658. 10.1038/s41598-021-90125-6 34017031 PMC8138002

[16] KelaiditiE CesariM CanevelliM van KanGA OussetPJ Gillette-GuyonnetS Cognitive frailty: rational and definition from an (I.A.N.A./I.A.G.G.) international consensus group. *J Nutr Health Aging.* (2013) 17:726–34. 10.1007/s12603-013-0367-2 24154642

[17] Ruan Q Yu Z Chen M Bao Z Li J He W. Cognitive frailty, a novel target for the prevention of elderly dependency. *Ageing Res Rev.* (2015) 20:1–10. 10.1016/j.arr.2014.12.004 25555677

[18] Panza F Lozupone M Solfrizzi V Stallone R Bellomo A Greco A Cognitive frailty: a potential target for secondary prevention of dementia. *Expert Opin Drug Metab Toxicol.* (2017) 13:1023–7. 10.1080/17425255.2017.1372424 28849681

[19] Bu Z Huang A Xue M Li Q Bai Y Xu G. Cognitive frailty as a predictor of adverse outcomes among older adults: A systematic review and meta-analysis. *Brain Behav.* (2021) 11:e01926. 10.1002/brb3.1926 33159430 PMC7821586

[20] Vatanabe IP Pedroso RV Teles RHG Ribeiro JC Manzine PR Pott-Junior H A systematic review and meta-analysis on cognitive frailty in community-dwelling older adults: risk and associated factors. *Aging Ment Health.* (2022) 26:464–76. 10.1080/13607863.2021.1884844 33612030

[21] Grande G Haaksma ML Rizzuto D Melis RJF Marengoni A Onder G Co-occurrence of cognitive impairment and physical frailty, and incidence of dementia: Systematic review and meta-analysis. *Neurosci Biobehav Rev.* (2019) 107:96–103. 10.1016/j.neubiorev.2019.09.001 31491474

[22] Page MJ McKenzie JE Bossuyt PM Boutron I Hoffmann TC Mulrow CD The PRISMA 2020 statement: an updated guideline for reporting systematic reviews. *BMJ.* (2021) 372:n71. 10.1136/bmj.n71 33782057 PMC8005924

[23] Stroup DF Berlin JA Morton SC Olkin I Williamson GD Rennie D Meta-analysis of observational studies in epidemiology: a proposal for reporting. Meta-analysis Of Observational Studies in Epidemiology (MOOSE) group. *JAMA.* (2000) 283:2008–12. 10.1001/jama.283.15.2008 10789670

[24] Wells G Shea B O’Connell D Peterson J Welch V Losos M *The Newcastle–Ottawa Scale (NOS) for assessing the quality if nonrandomized studies in meta-analyses.* (2021) Available online at: http://www.ohri.ca/programs/clinical_epidemiology/oxford.asp (accessed May 1, 2022).

[25] Egger M Davey Smith G Schneider M Minder C. Bias in meta-analysis detected by a simple, graphical test. *BMJ.* (1997) 315:629–34. 10.1136/bmj.315.7109.629 9310563 PMC2127453

[26] Downer B Al Snih S Howrey BT Raji MA Markides KS Ottenbacher KJ. Combined effects of cognitive impairment and pre-frailty on future frailty and death in older Mexican Americans. *Aging Ment Health.* (2019) 23:1405–12. 10.1080/13607863.2018.1493719 30472880 PMC6534489

[27] Avila-Funes JA Amieva H Barberger-Gateau P Le Goff M Raoux N Ritchie K Cognitive impairment improves the predictive validity of the phenotype of frailty for adverse health outcomes: the three-city study. *J Am Geriatr Soc.* (2009) 57:453–61. 10.1111/j.1532-5415.2008.02136.x 19245415

[28] Solfrizzi V Scafato E Seripa D Lozupone M Imbimbo BP D’Amato A Reversible cognitive frailty, dementia, and all-cause mortality. the Italian longitudinal study on aging. *J Am Med Dir Assoc.* (2017) 18:e1–89. 10.1016/j.jamda.2016.10.012 28012505

[29] Montero-Odasso MM Barnes B Speechley M Muir Hunter SW Doherty TJ Duque G Disentangling cognitive-frailty: results from the gait and brain study. *J Gerontol A Biol Sci Med Sci.* (2016) 71:1476–82. 10.1093/gerona/glw044 26984391

[30] Feng L Nyunt MS Gao Q Feng L Lee TS Tsoi T Physical frailty, cognitive impairment, and the risk of neurocognitive disorder in the Singapore longitudinal ageing studies. *J Gerontol Ser A Biol Sci Med Sci.* (2017) 72:369–75. 10.1093/gerona/glw050 27013397

[31] Feng L Zin Nyunt MS Gao Q Feng L Yap KB Ng TP. Cognitive frailty and adverse health outcomes: findings from the Singapore longitudinal ageing studies (SLAS). *J Am Med Dir Assoc.* (2017) 18:252–8. 10.1016/j.jamda.2016.09.015 27838339

[32] Yu R Morley JE Kwok T Leung J Cheung O Woo J. The effects of combinations of cognitive impairment and pre-frailty on adverse outcomes from a prospective community-based cohort study of older chinese people. *Front Med (Lausanne).* (2018) 5:50. 10.3389/fmed.2018.00050 29600247 PMC5863513

[33] Brigola AG Ottaviani AC Alexandre T da S Luchesi BM Pavarini SCI. Cumulative effects of cognitive impairment and frailty on functional decline, falls and hospitalization: A four-year follow-up study with older adults. *Arch Gerontol Geriatr.* (2020) 87:104005. 10.1016/j.archger.2019.104005 31901850

[34] Onder G Cesari M Maggio M Palmer K. Defining a care pathway for patients with multimorbidity or frailty. *Eur J Intern Med.* (2017) 38:1–2. 10.1016/j.ejim.2017.01.013 28111156

[35] Vetrano DL Rizzuto D Calderón-Larrañaga A Onder G Welmer AK Bernabei R Trajectories of functional decline in older adults with neuropsychiatric and cardiovascular multimorbidity: A Swedish cohort study. *PLoS Med.* (2018) 15:e1002503. 10.1371/journal.pmed.1002503 29509768 PMC5839531

[36] Zhang S Tomata Y Newson RB Sugawara Y Tsuji I. Combined healthy lifestyle behaviours and incident disability in an elderly population: the Ohsaki Cohort 2006 Study. *J Epidemiol Community Health.* (2018) 72:679–84. 10.1136/jech-2018-210464 29627784

[37] Shimada H Makizako H Doi T Tsutsumimoto K Lee S Suzuki T. Cognitive impairment and disability in older Japanese adults. *PLoS One.* (2016) 11:e0158720. 10.1371/journal.pone.0158720 27415430 PMC4945051

[38] Ofstedal MB Chiu CT Jagger C Saito Y Zimmer Z. Religion, life expectancy, and disability-free life expectancy among older women and men in the United States. *J Gerontol B Psychol Sci Soc Sci.* (2019) 74:e107–18. 10.1093/geronb/gby098 31585014 PMC6941211

[39] Boniolo G. Demented patients and the quandaries of identity: setting the problem, advancing a proposal. *Hist Philos Life Sci.* (2021) 43:21. 10.1007/s40656-021-00365-y 33587205 PMC7884352

[40] Wallace LMK Theou O Godin J Andrew MK Bennett DA Rockwood K. Investigation of frailty as a moderator of the relationship between neuropathology and dementia in Alzheimer’s disease: a cross-sectional analysis of data from the rush memory and aging project. *Lancet. Neurol.* (2019) 18:177–84. 10.1016/S1474-4422(18)30371-5 30663607 PMC11062500

[41] Chen TB Yiao SY Sun Y Lee HJ Yang SC Chiu MJ Comorbidity and dementia: a nationwide survey in Taiwan. *PLoS One.* (2017) 12:e0175475. 10.1371/journal.pone.0175475 28403222 PMC5389824

[42] Grasshoff J Beller J Kuhlmann BG Geyer S. Increasingly capable at the ripe old age? Cognitive abilities from 2004 to 2013 in Germany, Spain, and Sweden. *PLoS One.* (2021) 16:e0254038. 10.1371/journal.pone.0254038 34197534 PMC8248634

[43] Khaligh-Razavi SM Sadeghi M Khanbagi M Kalafatis C Nabavi SM. A self-administered, artificial intelligence (AI) platform for cognitive assessment in multiple sclerosis (MS). *BMC Neurol.* (2020) 20:193. 10.1186/s12883-020-01736-x 32423386 PMC7236354

[44] JacobsJM CohenA Ein-MorE MaaraviY StessmanJ. Frailty, cognitive impairment and mortality among the oldest old. *J Nutr Health Aging.* (2011) 15:678–82. 10.1007/s12603-011-0096-3 21968864

[45] St John PD Tyas SL Griffith LE Menec V. The cumulative effect of frailty and cognition on mortality – results of a prospective cohort study. *Int Psychogeriatr.* (2017) 29:535–43. 10.1017/S1041610216002088 27903307

[46] Del Brutto OH Mera RM Zambrano M Sedler MJ. Influence of frailty on cognitive decline: a population-based cohort study in rural ecuador. *J Am Med Dir Assoc.* (2019) 20:213–6. 10.1016/j.jamda.2018.09.023 30455048

[47] Sargent L Nalls M Starkweather A Hobgood S Thompson H Amella EJ Shared biological pathways for frailty and cognitive impairment: a systematic review. *Ageing Res Rev.* (2018) 47:149–58. 10.1016/j.arr.2018.08.001 30102995 PMC6376483

[48] Fabrício D de M Chagas MHN Diniz BS. Frailty and cognitive decline. *Transl Res.* (2020) 221:58–64. 10.1016/j.trsl.2020.01.002 32045578

[49] Baztán JJ De la Puente M Socorro A. Frailty, functional decline and mortality in hospitalized older adults. *Geriatr Gerontol Int.* (2017) 17:664–6. 10.1111/ggi.12925 28405967

[50] Junius-Walker U Onder G Soleymani D Wiese B Albaina O Bernabei R The essence of frailty: a systematic review and qualitative synthesis on frailty concepts and definitions. *Eur J Intern Med.* (2018) 56:3–10. 10.1016/j.ejim.2018.04.023 29861330

[51] Lee WJ Peng LN Liang CK Loh CH Chen LK. Cognitive frailty predicting all-cause mortality among community-living older adults in Taiwan: a 4-year nationwide population-based cohort study. *PLoS One.* (2018) 13:e0200447. 10.1371/journal.pone.0200447 30001354 PMC6042743

[52] Aliberti MJR Cenzer IS Smith AK Lee SJ Yaffe K Covinsky KE. Assessing risk for adverse outcomes in older adults: the need to include both physical frailty and cognition. *J Am Geriatr Soc.* (2019) 67:477–83. 10.1111/jgs.15683 30468258 PMC6510389

[53] Esteban-Cornejo I Cabanas-Sánchez V Higueras-Fresnillo S Ortega FB Kramer AF Rodriguez-Artalejo F Cognitive frailty and mortality in a national cohort of older adults: the role of physical activity. *Mayo Clin Proc.* (2019) 94:1180–9. 10.1016/j.mayocp 30871783

[54] OkuraM OgitaM AraiH. Self-reported cognitive frailty predicts adverse health outcomes for community-dwelling older adults based on an analysis of sex and age. *J Nutr Health Aging*. (2019) 23:654–64. 10.1007/s12603-019-1217-7 31367731

[55] Shimada H Doi T Lee S Makizako H Chen LK Arai H. Cognitive frailty predicts incident dementia among community-dwelling older people. *J Clin Med.* (2018a) 7(9):250. 10.3390/jcm7090250 30200236 PMC6162851

[56] ShimadaH MakizakoH TsutsumimotoK DoiT LeeS SuzukiT. Cognitive frailty and incidence of dementia in older persons. *J Prev Alzheimers Dis.* (2018b) 5:42–8. 10.14283/jpad.2017.29 29405232

[57] Solfrizzi V Scafato E Lozupone M Seripa D Giannini M Sardone R Additive role of a potentially reversible cognitive frailty model and inflammatory state on the risk of disability: the italian longitudinal study on aging. *Am J Geriatr Psychiatry.* (2017) 25:1236–48. 10.1016/j.jagp.2017.05.018 28689645

[58] TsutsumimotoK DoiT NakakuboS KimM KuritaS IshiiH Cognitive frailty as a risk factor for incident disability during late life: a 24-month follow-up longitudinal study. *J Nutr Health Aging.* (2020) 24:494–9. 10.1007/s12603-020-1365-9 32346687

