# Supplementary material for: Impact of frailty, mild cognitive impairment and cognitive frailty on adverse health outcomes among community-dwelling older adults: A systematic review and meta-analysis
Source: Front Med (Lausanne). 2022 Oct 31;9:1009794. doi: 10.3389/fmed.2022.1009794 (PMC9659908; doi:10.3389/fmed.2022.1009794)
Supplement: Supplementary file 2 [file Data_Sheet_2.PDF]

INPLASY PROTOCOL

To cite: Chen et al. Impact of cognitive frailty and physical frailty/cognitive impairment on adverse health outcomes among community-dwelling older adults: A systematic review and meta-analysis. Inplasy protocol 202250064. doi: 10.37766/inplasy2022.5.0064

Received: 11 May 2022

Published: 11 May 2022

Corresponding author:
Mengqian Li

mengqianli@ncu.edu.cn

Author Affiliation:
Department of Psychosomatic medicine, The First Affiliated Hospital of Nanchang University.

Support: **No financial support.**

Review Stage at time of this submission: **Data analysis.**

Conflicts of interest:
None declared.

Impact of cognitive frailty and physical frailty/cognitive impairment on adverse health outcomes among community-dwelling older adults: A systematic review and meta-analysis

Chen, BY[1]; He, Q[2]; Wang, MT[3]; Wang, Y[4]; Lai, XX[5]; Chen, HG[5]; Li, MQ[6].

Review question / Objective: **The purpose of this study is to analyze and compare association between the risk of adverse outcomes and participants with cognitive frailty and physical frailty alone, cognitive impairment alone in community-dwelling older adults by systematic review and meta-analysis. And it will discussion of the relationship between frailty and cognitive impairment, and the potential common mechanisms between them.**

Condition being studied: **Inclusion criteria: (a) prospective cohort studies or population-based longitudinal studies; (b) participants with 60 years old and older; (c) diagnostic criteria for physical frailty, cognitive impairment, and cognitive frailty need to be internationally agreed upon (e.g., frailty phenotype, frailty index, Minimum Mental State Examination); (d) hazard ratio (HR), odds ratio (OR), or risk ratio (RR) for the adverse outcomes (mortality, risk of dementia, hospitalization, disability) is reported, as well as outcomes or underlying data that contribute to the calculation of the above values. Exclusion criteria: (a) duplicate publications of the same data; (b) case reports, conference papers, reviews, systematic reviews, meta-analysis; (b) no relevant outcomes data (HR, OR, RR) available or insufficient statistics.**

INPLASY registration number: This protocol was registered with the International Platform of Registered Systematic Review and Meta-Analysis Protocols (INPLASY) on 11 May 2022 and was last updated on 11 May 2022 (registration number INPLASY202250064).

INTRODUCTION

Review question / Objective: **The purpose of this study is to analyze and compare association between the risk of adverse outcomes and participants with cognitive frailty and physical frailty alone, cognitive impairment alone in community-dwelling older adults by systematic review and meta-analysis. And it will discussion of the relationship between frailty and cognitive**

Chen et al. Inplasy protocol 202250064. doi:10.37766/inplasy2022.5.0064 Downloaded from https://inplasy.com/inplasy-2022-5-0064/

impairment, and the potential common mechanisms between them.

Rationale: Aging is a global, spontaneous, progressive, inevitable process of biology over time, which is characterized by degenerative changes of structure and decline of function, weakening of adaptability and resistance, as well as the loss of tissue cells and constituent substances. It can result in deficits on physical and mental, such as physical frailty and cognitive impairment. These two processes will lead to higher rates of adverse outcomes, faster rates of disability, and shorter survival periods in older adults. In the later stages of life, physical and cognitive problems often emerge simultaneously and interact with each other to produce a complex health state. And recent epidemiological studies also point out that physical frailty increases the risk of cognitive decline and vice versa. Thus, cognitive frailty has been proposed since to predict adverse health outcomes in older adults.

Condition being studied: Frailty is a key intermediate state in the aging process and is a dynamic process. Frailty has been described as a multidimensional clinical gerontological syndrome, characterized by cumulative decline of multiple physiological systems that leads to reduced energy reserves and increased susceptibility to stressors, and the dysregulated of physiological system dynamic balance. It can lead to a variety of adverse outcomes, such as mortality, dementia, disability and hospitalization, seriously affecting the quality of life of the elderly. Frailty and cognitive impairment are usually regarded as two separate concepts, but a growing body of research have confirmed that cognitive impairment is significantly associated with physical frailty, as cognitive impairment and physical frailty tend to coexist and interact with each other in later life, mutually contributing to their negative impact on health. When they coexist, cumulative negative effects are often detected, significantly increasing mortality or other adverse outcomes. Although epidemiological and clinical studies suggest a strong association between frailty and cognitive impairment, the specific mechanisms have not been elucidated. Recently cognitive frailty has been proposed to coexist with physical frailty and cognitive impairment, but without dementia, and studies have demonstrated that this syndrome increases the risk of adverse health outcomes.

METHODS

Search strategy: We developed a specific search strategy for each electronic database and searched PubMed, Web of Science, MEDLINE, Embase, and collected original studies on the relationship between cognitive impairment, physical frailty, cognitive frailty, and adverse health outcomes in older adults.

Participant or population: 60 years old and older in community-dwelling older adults.

Intervention: Exposure: Includes older participants with cognitive frailty, only physical frailty (or pre-frailty), and only cognitive impairment without dementia.

Comparator: Non-exposure: Robust older adults without cognitive impairment or physical frailty.

Study designs to be included: Prospective cohort studies or population-based longitudinal studies were included in this review.

Eligibility criteria: Inclusion criteria: (a) prospective cohort studies or population-based longitudinal studies; (b) participants with 60 years old and older; (c) diagnostic criteria for physical frailty, cognitive impairment, and cognitive frailty need to be internationally agreed upon (e.g., frailty phenotype, frailty index, Minimum Mental State Examination); (d) hazard ratio (HR), odds ratio (OR), or risk ratio (RR) for the adverse outcomes (mortality, risk of dementia, hospitalization, disability) is reported, as well as outcomes or underlying data that contribute to the calculation of the above values. Exclusion criteria: (a) duplicate publications of the

Chen et al. Inplasy protocol 202250064. doi:10.37766/inplasy2022.5.0064 Downloaded from https://inplasy.com/inplasy-2022-5-0064/

same data; (b) case reports, conference papers, reviews, systematic reviews, meta-analysis; (b) no relevant outcomes data (HR, OR, RR) available or insufficient statistics.

Information sources: PubMed, Web of Science, MEDLINE, Embase were used as the primary data sources, and the search period ranged from the initial to July 2020, while reference lists of existing reviews and meta-analyses were also manually screened.

Main outcome(s): Primary outcomes include the impact of cognitive frailty, physical frailty, and cognitive impairment on the risk of mortality, dementia, disability, and hospitalization.

Additional outcome(s): Secondary outcomes included the effects of cognitive frailty, physical frailty, cognitive impairment on quality of life, falls, nursing home admission.

Data management: We used Microsoft Excel to record the information in the included articles.

Quality assessment / Risk of bias analysis: Quality assessment was performed using the Newcastle-Ottawa scale (NOS).

Strategy of data synthesis: Two investigators independently extracted the basic characteristics of the included studies and the hazard ratios (HR)/odds ratios (OR)/risk ratios (RR) with 95 % confidence interval (CI) of each study for the association of cognitive frailty/physical frailty/cognitive impairment with adverse outcomes (mortality/dementia/disability/hospitalization). The Q test and the I2 statistics would be used to assess the heterogeneity of the included studies, where p>0.1 of the Q statistics and I2 value 50%. Otherwise used fixed model. Publication bias funnel plots were conducted to detect the publication bias. A symmetrical funnel plot is likely to indicate low publication bias while an asymmetric funnel plot is likely to indicate publication bias. All statistical analyses were performed using Review Manager (RevMan) 5.3 software.

Subgroup analysis: Subgroup analyses were performed based on different adverse health outcomes and the presence of cognitive frailty, physical frailty only (or pre-frailty), and cognitive impairment only in participants to identify the indicator that had the greatest impact on adverse outcomes in older adults.

Sensitivity analysis: The sensitivity of the article is reflected by the change in the effect size after the removal of an article in included articles and the stability of the detection results.

Language: Only English.

Country(ies) involved: China.

Keywords: Cognitive frailty; Frailty; Cognitive impairment; Adverse outcomes; Older adults; Mortality; Dementia; Disability; Hospitalization; Falls; Quality of life.

Contributions of each author:

Author 1 - Baoyu Chen.
Email: cby810@hotmail.com
Author 2 - Qin He.
Email: 411439420001@email.ncu.edu.cn
Author 3 - Mingting Wang.
Email: 411439420004@email.ncu.edu.cn
Author 4 - Yong Wang.
Email: wangyong751006@163.com
Author 5 - Xiaoxing Lai.
Email: laixx86@163.com
Author 6 - Hongguang Chen.
Email: chenhg@bjmu.edu.cn
Author 7 - Mengqian Li.
Email: engqianli@ncu.edu.cn

Chen et al. Inplasy protocol 202250064. doi:10.37766/inplasy2022.5.0064 Downloaded from https://inplasy.com/inplasy-2022-5-0064/
