# Supplementary material for: Impact of frailty, mild cognitive impairment and cognitive frailty on adverse health outcomes among community-dwelling older adults: A systematic review and meta-analysis
Source: Front Med (Lausanne). 2022 Oct 31;9:1009794. doi: 10.3389/fmed.2022.1009794 (PMC9659908; doi:10.3389/fmed.2022.1009794)
Supplement: Supplementary file 3 [file Data_Sheet_3.docx]

**Title:** Impact of frailty, mild cognitive impairment and cognitive frailty on adverse health outcomes among community-dwelling older adults: A systematic review and meta-analysis

**Supplementary Data**

**S1**: Search strategy

**Table 1**: Quality evaluation of included studies using the Newcastle-Ottawa Scale (NOS)

**Fig 1**: Pooled RRs for individuals with cognitive frailty, frailty and mild cognitive impairment with adverse outcomes.

**Fig 2**: Forest plot of the association between mild cognitive impairment with pre-frailty, pre-frailty and mortality in older adults.

**Fig 3**: Forest plot of the association between mild cognitive impairment with pre-frailty, pre-frailty and dementia in older adults.

**Fig 4**: Forest plot of the association between mild cognitive impairment with pre-frailty and hospitalization in older adults.

**Fig 5**: Funnel plot for the effect of cognitive frailty, frailty, and mild cognitive impairment on mortality.

**Fig 6:** Funnel plot for the effect of cognitive frailty, frailty, and mild cognitive impairment on dementia.

**Fig 7**: Funnel plot of the effect of cognitive frailty, frailty, and mild cognitive impairment on hospitalization.

**Fig 8**: Funnel plot of the effect of cognitive frailty, frailty, and mild cognitive impairment on disability.

**M1**: Included literatures in this systematic review and meta-analysis.

**S1: Search strategy**

**Medline（n=638）**

AB((cognitive aging) OR (cognitive dysfunction) OR (Dysfunction Cognitive) OR (Cognitive Impairment) OR (Mild Cognitive Impairment) OR (Mild Neurocognitive Disorder) OR (Cognitive Decline) OR (Mental Deterioration) OR (dementia) OR (Amentia) OR (Senile Paranoid Dementia) OR (Familial Dementia) OR (Alzheimer OR (Alzheimer Dementias) OR (Alzheimer Sclerosis) AND AB((frailty) OR (Frailties OR (Frailness) OR (Frailty Syndrome) OR (Debility) OR (Debilities)) AND AB((elderly) OR (elder) OR (geriatrics)) OR (Aged) AND AB((healthy outcomes) OR (Cognitive-related outcomes) OR (Lifestyle)) AND AB (functional disability) AND AB ((Mortality) OR (Mortalities) OR（Case Fatality Rates) OR (Crude Death Rate) OR (Crude Mortality Rate) OR (Death rate) OR (Mortality rate) OR (Excess Mortality) OR (Age-Specific Death Rate) OR (life quality)) AND AB ((hospitalization) OR (hospitalizations)) AND AB ((Quality of life) OR (Lifestyles) OR (Lifestyle) OR (Life Style induced illness) OR (Lifestyle factors) OR (Lifestyle factor))

**Embase（n=2426）**

(cognitive AND aging OR (cognitive AND dysfunction) OR (dysfunction, AND cognitive) OR (cognitive AND impairment) OR (mild AND cognitive AND impairment) OR (mild AND neurocognitive AND disorder) OR (cognitive AND decline) OR (mental AND deterioration) OR dementia OR amentia OR (senile AND paranoid AND dementia) OR (familial AND dementia) OR Alzheimer OR (Alzheimer AND dementias) OR (Alzheimer AND sclerosis)) AND (frailty OR frailties OR frailness OR (frailty AND syndrome) OR debility OR debilities) AND (elderly OR elder OR (older AND adults) OR geriatrics OR aged) AND (healthy AND outcomes OR (cognitive related AND outcomes) OR (factor. AND lifestyle OR lifestyle OR lifestyles OR (life AND style AND induced AND illness) OR (lifestyle AND factors) OR (lifestyle AND factor) OR mortality OR mortalities OR (case AND fatality AND rates) OR (functional AND disability) OR hospitalization OR hospitalizations OR (quality AND of AND life) OR (crude AND death AND rate) OR (crude AND mortality AND rate) OR (death AND rate) OR (excess AND mortality) OR (mortality AND decline) OR ('age specific' AND death AND rate) OR (life AND quality) OR ('health related’ AND quality AND of AND life) OR hrqol)

**Web of Science (n=2755）**

①: Ts= ((cognitive aging) OR (cognitive dysfunction) OR (Dysfunction, Cognitive) OR (Cognitive Impairment) OR (Mild Cognitive Impairment) OR (Mild Neurocognitive Disorder) OR (Cognitive Decline) OR (Mental Deterioration) OR (dementia) OR (Amentia) OR (Senile Paranoid Dementia) OR (Familial Dementia) OR (Alzheimer) OR (Alzheimer Dementias) OR (Alzheimer Sclerosis))

②: TS=((frailty) OR (Frailties) OR (Frailness) OR (Frailty Syndrome) OR (Debility) OR (Debilities))

③: Ts=((elderly) OR (elder) OR (older adults) OR (geriatrics) OR (Aged))

④: Ts=((healthy outcomes) OR (cognitive-related outcomes) OR (lifestyle) OR (Factor, Lifestyle) OR (Lifestyle) OR (Lifestyles) OR (Life Style Induced Illness) OR (Lifestyle Factors) OR (Lifestyle Factor) OR (Mortality) OR (Mortalities) OR (Case Fatality Rates) OR (functional disability) OR (hospitalization) OR (Hospitalizations) OR (quality of life) OR (Crude Death Rate) OR (Crude Mortality Rate) OR (Death Rate) OR (Excess Mortality) OR (Mortality Decline) OR (Age-Specific Death Rate) OR (Life Quality) OR (Health-Related Quality Of Life))

⑤:①#②#③#④

**PubMed (n=565）**

(((((((((((((((cognitive aging[Title/Abstract]) OR (cognitive dysfunction[Title/Abstract])) OR (dysfunction,cognitive[Title/Abstract])) OR (cognitive impairment[Title/Abstract])) OR (mild cognitive impairment[Title/Abstract])) OR (mild neurocognitive disorder[Title/Abstract])) OR (cognitive decline[Title/Abstract])) OR (dementia[Title/Abstract])) OR (senile paranoid dementia[Title/Abstract])) OR (familial dementia[Title/Abstract])) OR (Alzheimer[Title/Abstract])) OR (Alzheimer dementias[Title/Abstract])) OR (Alzheimer sclerosis[Title/Abstract])) AND ((((((frailty[Title/Abstract]) OR (frailties[Title/Abstract])) OR (frailness[Title/Abstract])) OR (frailty syndrome[Title/Abstract])) OR (debility[Title/Abstract])) OR (debilities[Title/Abstract]))) AND ((((elderly[Title/Abstract]) OR (older adults[Title/Abstract])) OR (geriatrics[Title/Abstract])) OR (aged[Title/Abstract]))) AND (((((((((((((((((((((((((healthy outcomes[Title/Abstract]) OR (cognitive-related outcomes[Title/Abstract])) OR (lifestyle[Title/Abstract])) OR (functional disability[Title/Abstract])) OR (mortality[Title/Abstract])) OR (mortalities[Title/Abstract])) OR (case fatality rates[Title/Abstract])) OR (crude death rate[Title/Abstract])) OR (crude mortality rate[Title/Abstract])) OR (death rate[Title/Abstract])) OR (mortality rate[Title/Abstract])) OR (excess mortality[Title/Abstract])) OR (mortality decline[Title/Abstract])) OR (age-specific death rate[Title/Abstract])) OR (life quality[Title/Abstract])) OR (life quality[Title/Abstract])) OR (hospitalization[Title/Abstract])) OR (hospitalizations[Title/Abstract])) OR (quality of life[Title/Abstract])) OR (lifestyle[Title/Abstract])) OR (lifestyles[Title/Abstract])) OR (life style induced illness[Title/Abstract])) OR (lifestyle factors[Title/Abstract])) OR (factor, lifestyle[Title/Abstract])) OR (lifestyle factor[Title/Abstract]))

**Table 1: Quality evaluation of included studies using the Newcastle-Ottawa Scale (NOS)**

| **Study** | **Selection** | | | |  | **Comparability** | |  | **Outcome** | | | **Total score** |
| --- | --- | --- | --- | --- | --- | --- | --- | --- | --- | --- | --- | --- |
|  | Representativeness of the exposed cohort | Selection of the nonexposed cohort | Ascertainment of exposure | Demonstration that outcome of interest was not present at start of study |  | According the most important factor to choose control | According the other important factor to choose control |  | Assessment of outcome | Follow-up long enough for outcome to occur | Adequacy of follow- up of cohorts |  |
| Aliberti et al. 2019 | ☆ | ☆ | ☆ | ☆ |  | ☆ | ☆ |  | ☆ | ☆ | ☆ | 9 |
| Avila-Funes et al.2009 | ☆ | ☆ | ☆ |  |  | ☆ | ☆ |  | ☆ |  | ☆ | 7 |
| Brigola et al. 2020 | ☆ | ☆ | ☆ | ☆ |  |  | ☆ |  | ☆ |  | ☆ | 7 |
| Downer et al. 2019 | ☆ | ☆ | ☆ | ☆ |  | ☆ | ☆ |  | ☆ | ☆ | ☆ | 9 |
| Downer et al. 2020 | ☆ | ☆ | ☆ | ☆ |  | ☆ | ☆ |  | ☆ | ☆ | ☆ | 9 |
| Esteban-Cornejo et al. 2019 | ☆ | ☆ | ☆ | ☆ |  |  | ☆ |  | ☆ | ☆ | ☆ | 8 |
| Feng et al. 2017a | ☆ | ☆ | ☆ |  |  | ☆ | ☆ |  | ☆ |  | ☆ | 7 |
| Feng et al. 2017b | ☆ | ☆ | ☆ | ☆ |  | ☆ | ☆ |  | ☆ |  |  | 7 |
| Hao et al. 2018 |  | ☆ | ☆ | ☆ |  | ☆ | ☆ |  | ☆ |  | ☆ | 7 |
| Lee WJ et al. 2018 | ☆ | ☆ | ☆ | ☆ |  | ☆ | ☆ |  | ☆ |  | ☆ | 8 |
| Lee Y et al. 2018 | ☆ | ☆ | ☆ | ☆ |  | ☆ | ☆ |  | ☆ |  | ☆ | 8 |
| Liu LK et al. 2018 | ☆ | ☆ | ☆ | ☆ |  |  | ☆ |  | ☆ |  | ☆ | 7 |
| Liu Z et al. 2018 | ☆ | ☆ | ☆ | ☆ |  | ☆ | ☆ |  | ☆ | ☆ | ☆ | 9 |
| Montero-Odasso et al. 2016 |  | ☆ | ☆ | ☆ |  |  | ☆ |  | ☆ | ☆ | ☆ | 7 |
| Okura et al. 2019 | ☆ | ☆ |  | ☆ |  | ☆ | ☆ |  | ☆ |  | ☆ | 7 |
| Shimada et al. 2018a | ☆ | ☆ | ☆ | ☆ |  | ☆ | ☆ |  | ☆ |  | ☆ | 8 |
| Shimada et al. 2018b | ☆ | ☆ | ☆ | ☆ |  | ☆ | ☆ |  | ☆ |  | ☆ | 8 |
| Solfrizzi et al. 2017a | ☆ | ☆ | ☆ |  |  | ☆ | ☆ |  | ☆ |  | ☆ | 7 |
| Solfrizzi et al. 2017b | ☆ | ☆ | ☆ | ☆ |  | ☆ | ☆ |  | ☆ | ☆ | ☆ | 9 |
| St John et al.2017 | ☆ | ☆ | ☆ |  |  |  | ☆ |  | ☆ | ☆ | ☆ | 7 |
| Tsutsumimo et al. 2020 | ☆ | ☆ | ☆ | ☆ |  | ☆ | ☆ |  | ☆ |  | ☆ | 8 |
| Yu et al. 2018 | ☆ | ☆ | ☆ | ☆ |  | ☆ | ☆ |  | ☆ | ☆ | ☆ | 9 |

**Fig 1:** Pooled RRs for individuals with cognitive frailty, frailty, and mild cognitive impairment with adverse outcomes.


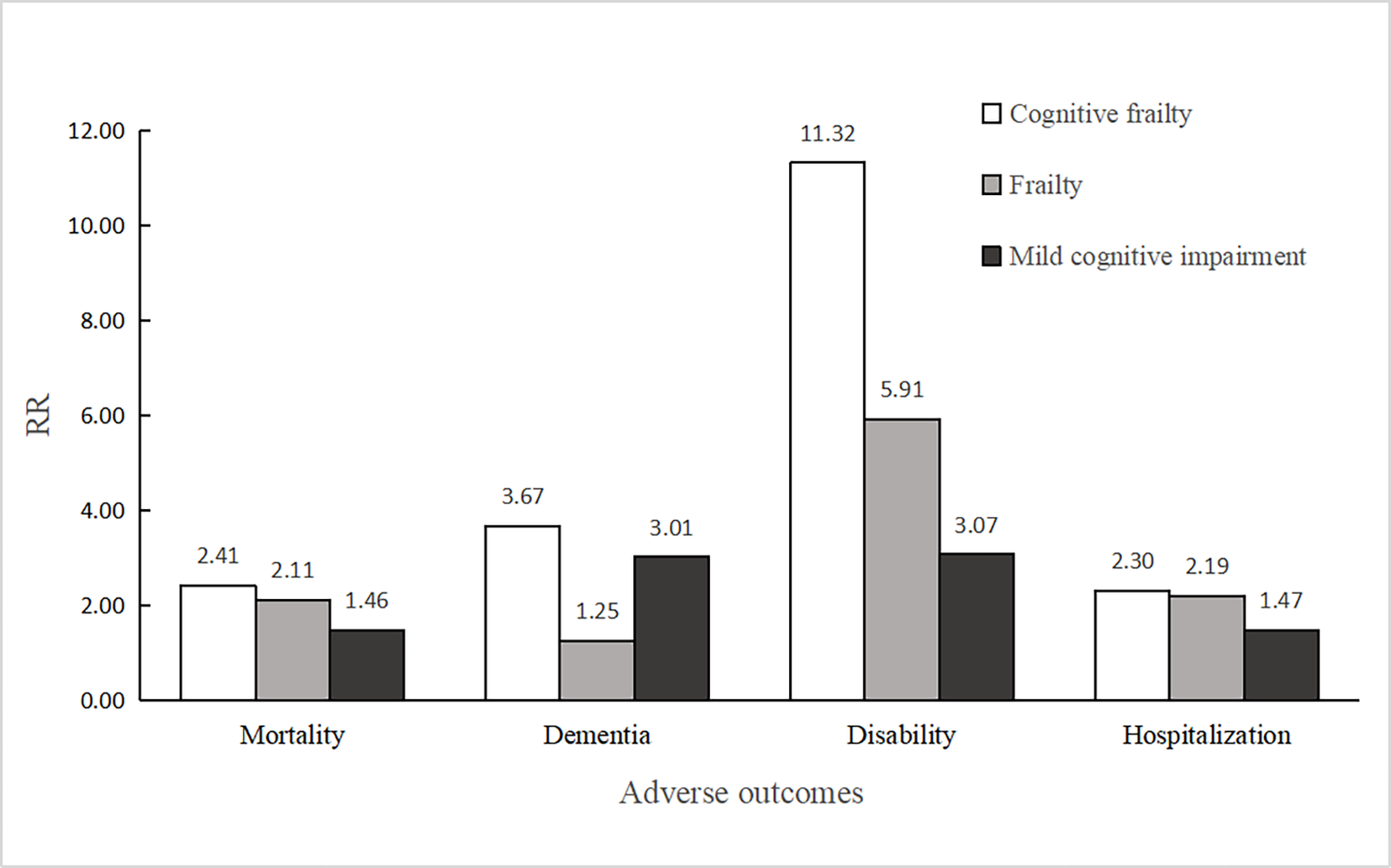


**Fig 2**: **Mortality**


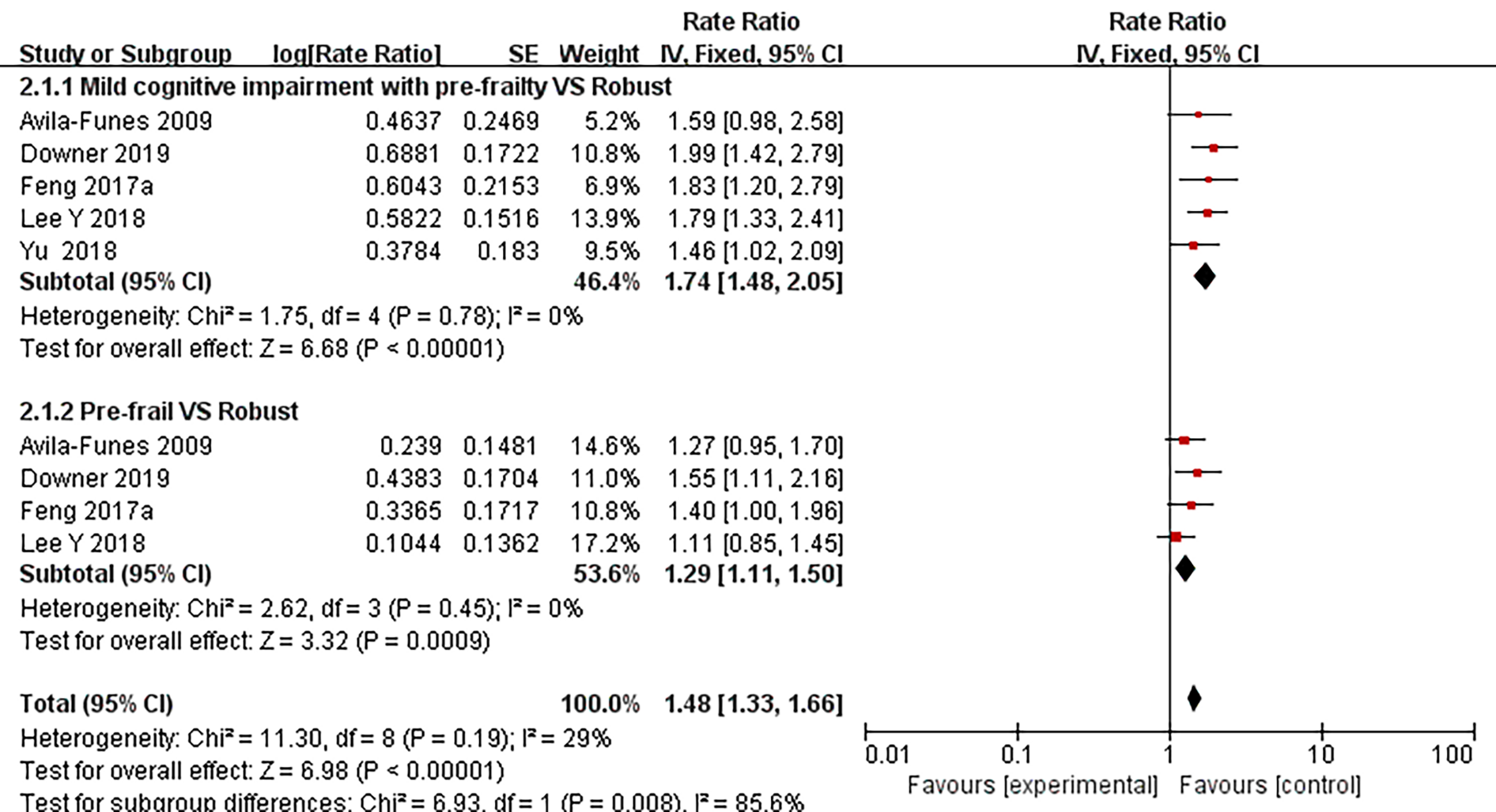


Fig 2. Forest plot of the association between mild cognitive impairment with pre-frailty, pre-frailty and mortality in older adults (reference group: participants were free of frailty and mild cognitive impairment), using fixed-effects meta-analysis. 95%CI: 95% confidence interval.

**Fig 3: Dementia**


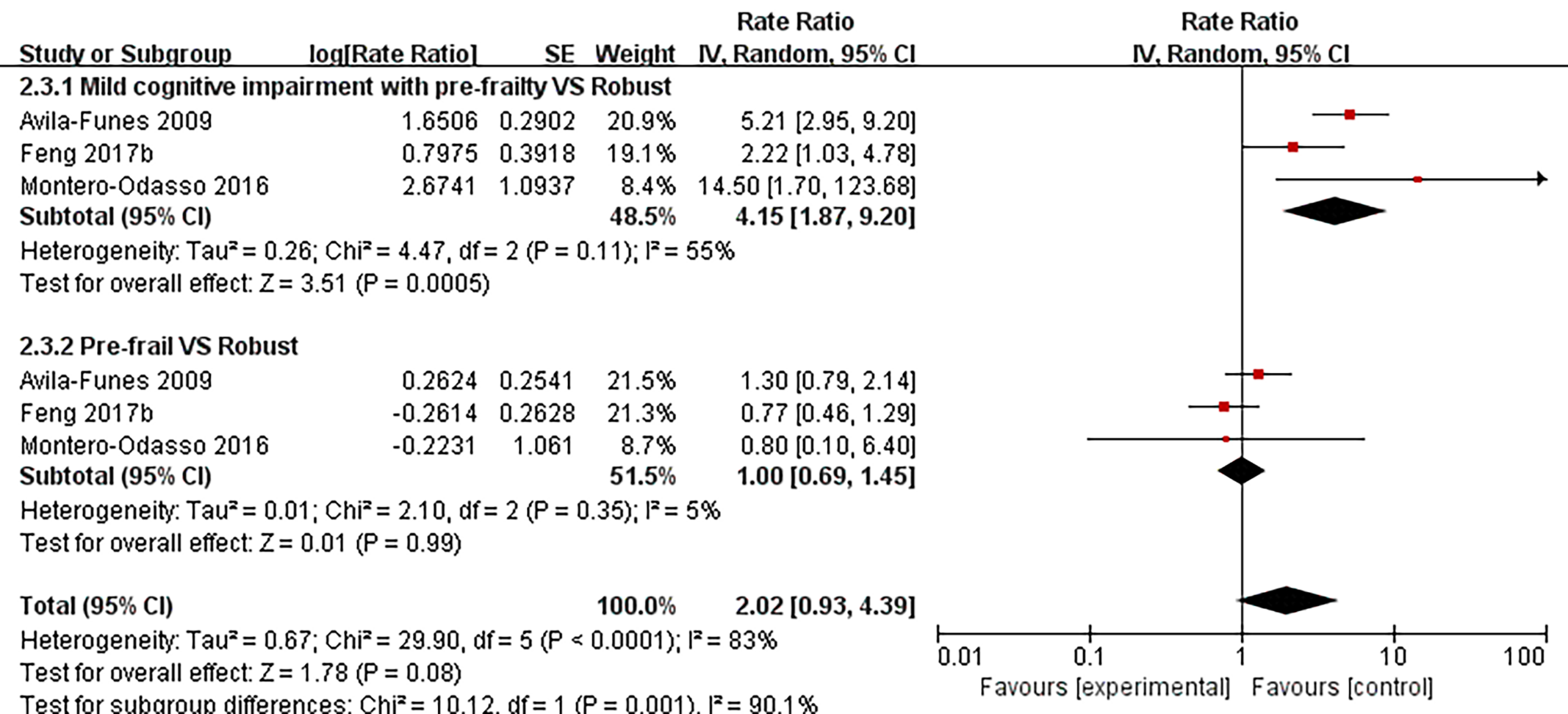


Fig 3. Forest plot of the association between mild cognitive impairment with pre-frailty, pre-frailty and incident dementia in older adults (reference group: participants were free of frailty and mild cognitive impairment), using random-effects meta-analysis. 95%CI: 95% confidence interval.

**Fig 4: Hospitalization**


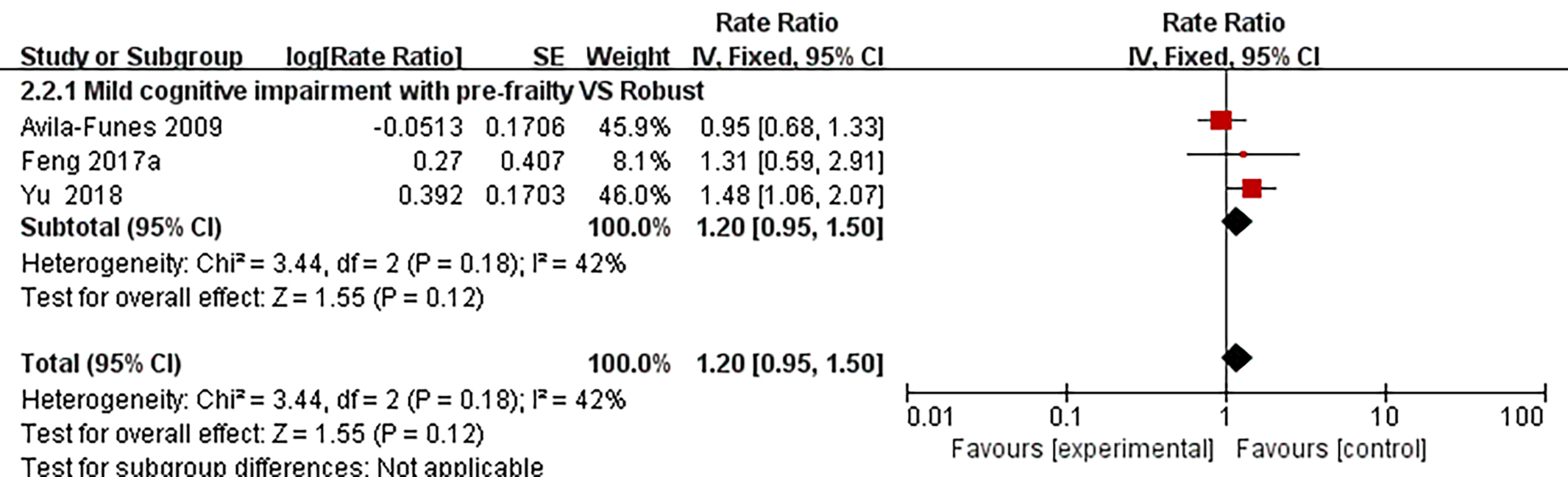


Fig 4: Forest plot of the association between mild cognitive impairment with pre-frailty and hospitalization in older adults (reference group: participants were free of frailty and mild cognitive impairment), using fixed-effects meta-analysis. 95%CI: 95% confidence interval.

**Fig 5: Funnel plot for mortality**

**
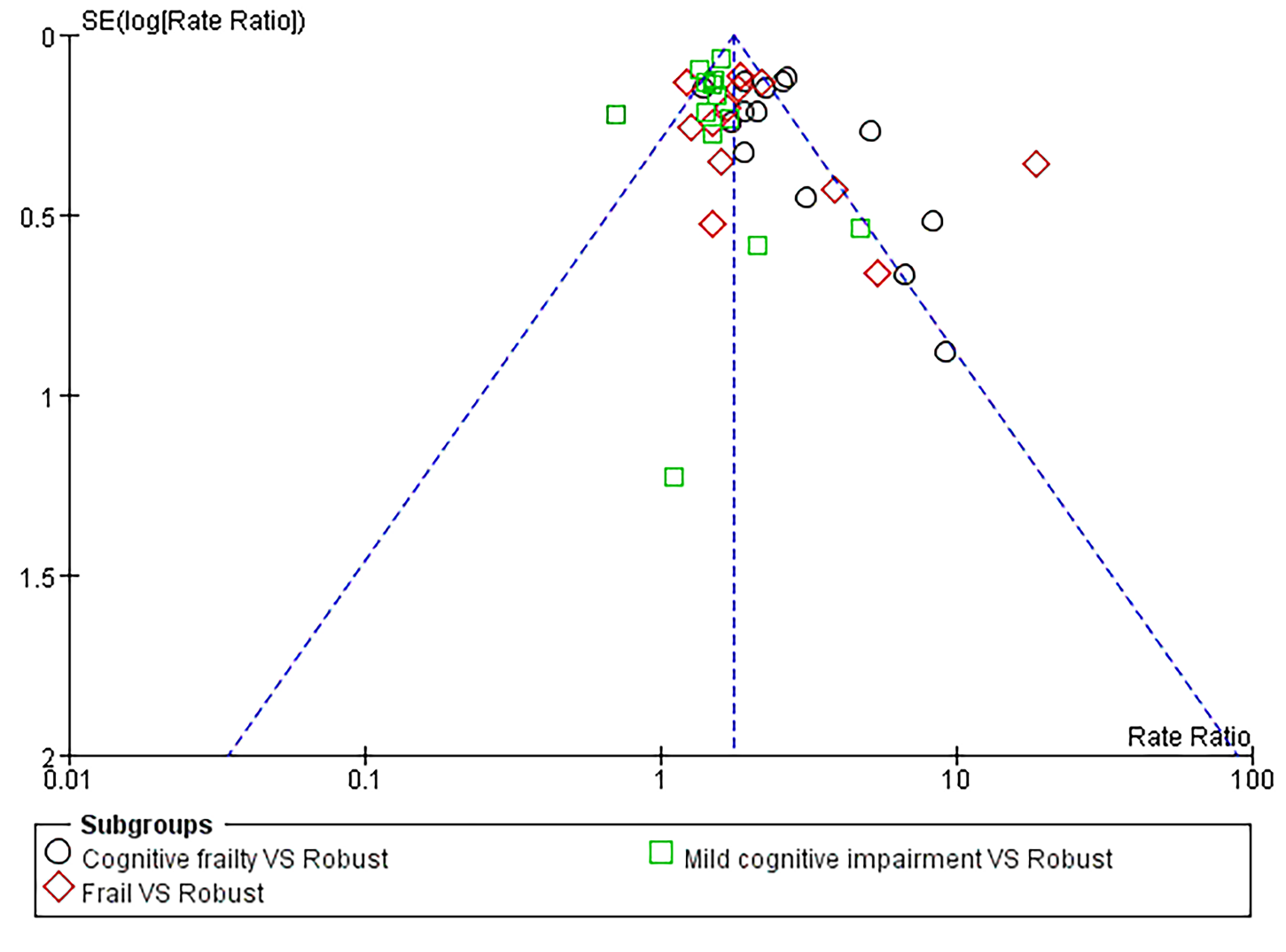
**

Fig 5: Funnel plot for the effect of cognitive frailty, frailty, and mild cognitive impairment on mortality.

**Fig 6: Funnel plot for dementia**

**
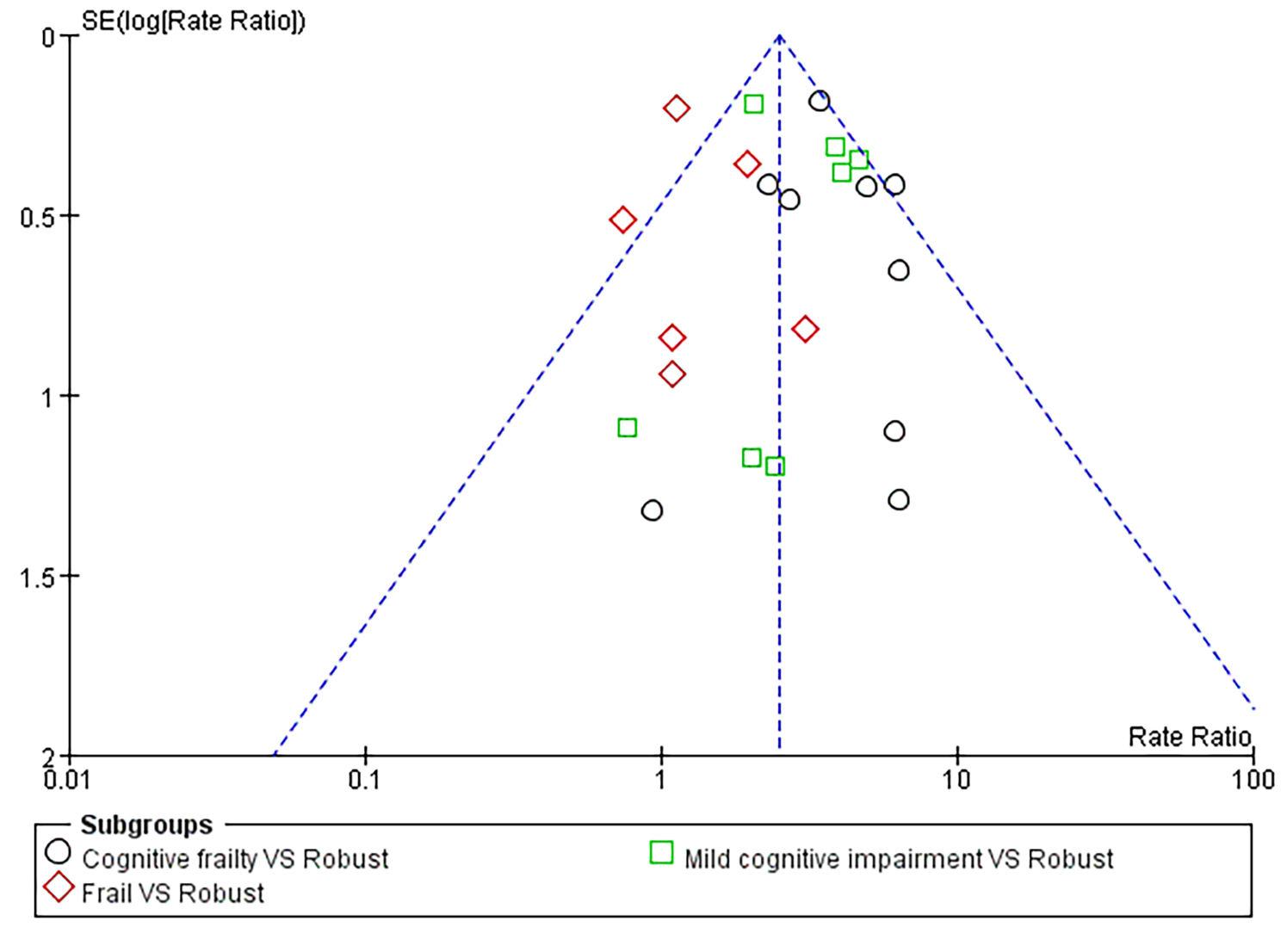
**

Fig 6: Funnel plot for the effect of cognitive frailty, frailty, and mild cognitive impairment on dementia.

**Fig 7: Funnel plot for hospitalization**

**
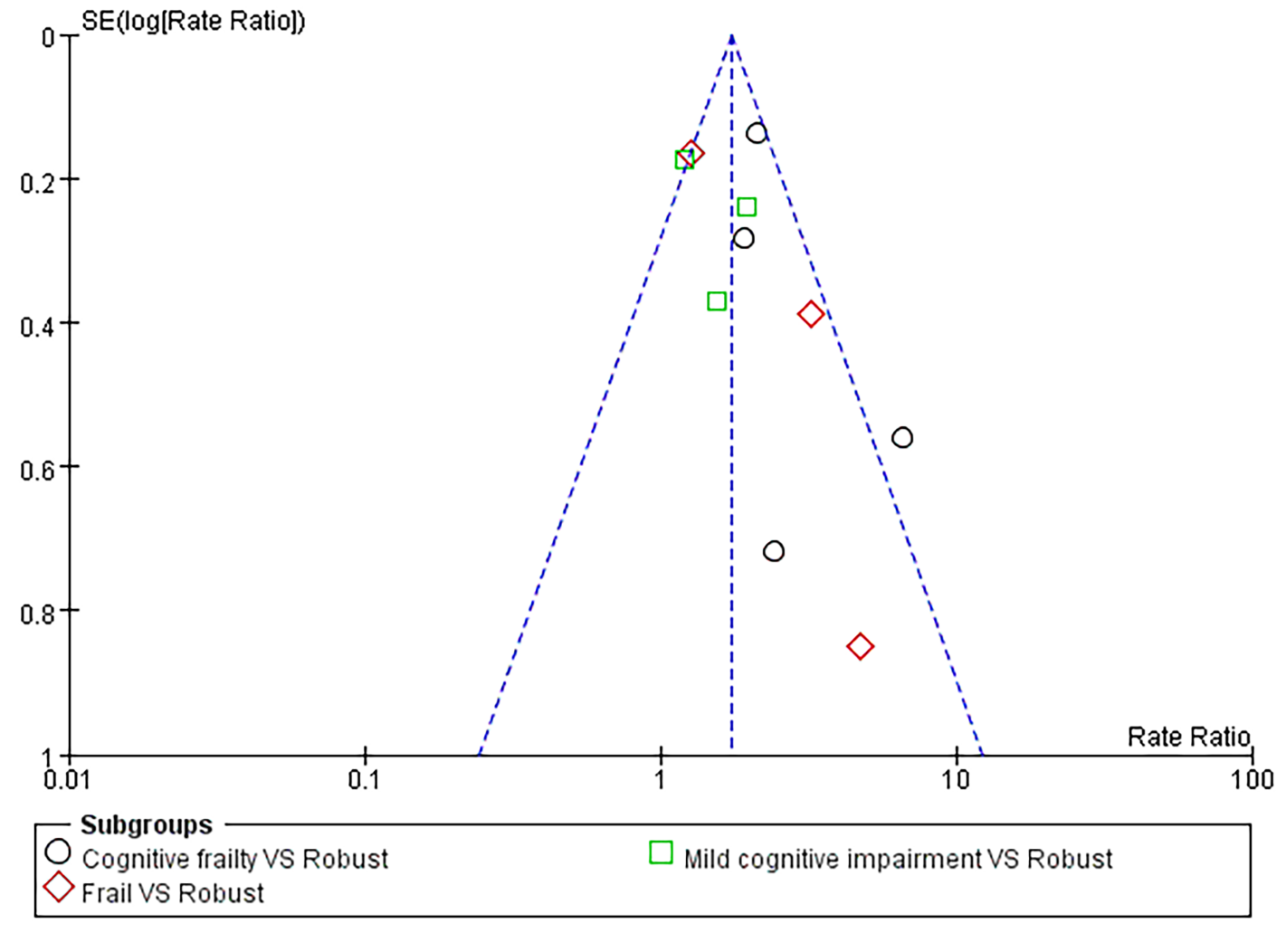
**

Fig 7: Funnel plot of the effect of cognitive frailty, frailty, and mild cognitive impairment on hospitalization.

**Fig 8: Funnel plot for disability**

**
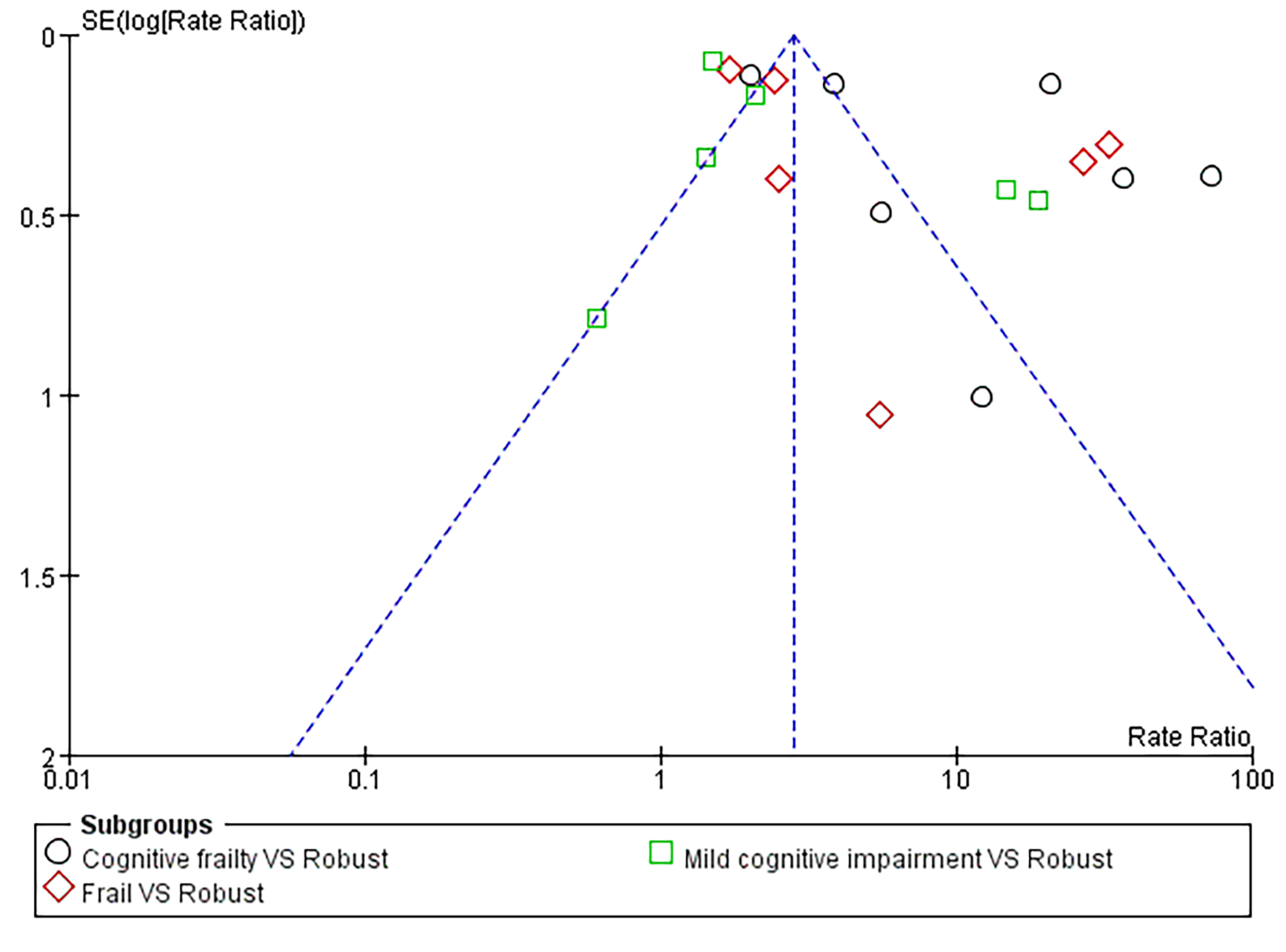
**

Fig 8: Funnel plot of the effect of cognitive frailty, frailty, and mild cognitive impairment on disability.

**M1: All included studies in this systematic review and meta-analysis**

1. Aliberti MJR, Cenzer IS, Smith AK, Lee SJ, Yaffe K, Covinsky KE. Assessing Risk for Adverse Outcomes in Older Adults: The Need to Include Both Physical Frailty and Cognition. *J Am Geriatr* Soc 2019; **67**: 477–83.

2. Avila-Funes JA, Amieva H, Barberger-Gateau P et al. Cognitive impairment improves the predictive validity of the phenotype of frailty for adverse health outcomes: the three-city study. *J Am Geriatr Soc* 2009; **57**: 453–61.

3. Brigola AG, Ottaviani AC, Alexandre TDS, Luchesi BM, Pavarini SCI. Cumulative effects of cognitive impairment and frailty on functional decline, falls and hospitalization: A four-year follow-up study with older adults. *Arch Gerontol Geriatr* 2020; **87**: 104005.

4. Downer B, Al Snih S, Howrey BT, Raji MA, Markides KS, Ottenbacher KJ. Combined effects of cognitive impairment and pre-frailty on future frailty and death in older Mexican Americans. *Aging Ment Health* 2019; **23**: 1405–12.

5. Downer B, Milani S, Wong R. The Sequence of Physical and Cognitive Impairment and the Association with Mortality Among Unimpaired Older Mexican Adults. *J Gerontol A Biol Sci Med Sci* 2020; **75**: 1386–92.

6. Esteban-Cornejo I, Cabanas-Sánchez V, Higueras-Fresnillo S et al. Cognitive Frailty and Mortality in a National Cohort of Older Adults: the Role of Physical Activity. *Mayo Clin Proc* 2019; **94**: 1180–9.

7. Feng L, Zin Nyunt MS, Gao Q, Feng L, Yap KB, Ng TP. Cognitive Frailty and Adverse Health Outcomes: Findings From the Singapore Longitudinal Ageing Studies (SLAS). *J Am Med Dir Assoc* 2017; **18**: 252–8.

8. Feng L, Nyunt MS, Gao Q, et al. Physical Frailty, Cognitive Impairment, and the Risk of Neurocognitive Disorder in the Singapore Longitudinal Ageing Studies. *J Gerontol A Biol Sci Med Sci* 2017; **72**: 369-75.

9. Hao Q, Dong B, Yang M et al. Frailty and Cognitive Impairment in Predicting Mortality Among Oldest-Old People. *Front Aging Neurosci* 2018; **10**: 295.

10. Lee WJ, Peng LN, Liang CK, Loh CH, Chen LK. Cognitive frailty predicting all-cause mortality among community-living older adults in Taiwan: A 4-year nationwide population-based cohort study. *PLoS One* 2018; **13**: e0200447.

11. Lee Y, Kim J, Chon D et al. The effects of frailty and cognitive impairment on 3-year mortality in older adults. *Maturitas* 2018; **107**: 50–55.

12. Liu L-K, Chen C-H, Lee W-J et al. Cognitive Frailty and Its Association with All-Cause Mortality Among Community-Dwelling Older Adults in Taiwan: Results from I-Lan Longitudinal Aging Study. *Rejuvenation Res* 2018; **21**: 510–7.

13.Liu Z, Han L, Gahbauer EA, Allore HG, Gill TM. Joint Trajectories of Cognition and Frailty and Associated Burden of Patient-Reported Outcomes. *J Am Med Dir Assoc* 2018; **19**: 304-9.e2.

14. Montero-Odasso MM, Barnes B, Speechley M et al. Disentangling Cognitive-Frailty: Results From the Gait and Brain Study. *J Gerontol A Biol Sci Med Sci* 2016; **71**: 1476-1482.

15. Okura M, Ogita M, Arai H.. Self-Reported Cognitive Frailty Predicts Adverse Health Outcomes for Community-Dwelling Older Adults Based on an Analysis of Sex and Age. *J Nutr Health Aging* 2019; **23**: 654-664.

16. Shimada H, Doi T, Lee S, Makizako H, Chen LK, Arai H. Cognitive Frailty Predicts Incident Dementia among Community-Dwelling Older People. *J Clin Med* 2018; **7**: 250.

17. Shimada H, Makizako H, Tsutsumimoto K, Doi T, Lee S, Suzuki T. Cognitive Frailty and Incidence of Dementia in Older Persons. *J Prev Alzheimers Dis* 2018; **5**: 42-48.

18. Solfrizzi V, Scafato E, Lozupone M et al. Additive Role of a Potentially Reversible Cognitive Frailty Model and Inflammatory State on the Risk of Disability: The Italian Longitudinal Study on Aging. *Am J Geriatr Psychiatry* 2017; **25**: 1236–48.

19. Solfrizzi V, Scafato E, Seripa D et al. Reversible Cognitive Frailty, Dementia, and All-Cause Mortality. The Italian Longitudinal Study on Aging. *J Am Med Dir Assoc* 2017; **18**: 89.e1-89.e8.

20. St John PD, Tyas SL, Griffith LE, Menec V. The cumulative effect of frailty and cognition on mortality - results of a prospective cohort study. *Int Psychogeriatr* 2017; **29**: 535–43.

21. Tsutsumimoto K, Doi T, Nakakubo S et al. Cognitive Frailty as a Risk Factor for Incident Disability During Late Life: A 24-Month Follow-Up Longitudinal Study. *J Nutr Health Aging* 2020; **24**: 494–9.

22. Yu R, Morley JE, Kwok T, Leung J, Cheung O, Woo J. The Effects of Combinations of Cognitive Impairment and Pre-frailty on Adverse Outcomes from a Prospective Community-Based Cohort Study of Older Chinese People. *Front Med (Lausanne)* 2018; **5**: 50.
